# Integrated mRNA and miRNA Transcriptomic Analyses Reveals Divergent Mechanisms of Sunitinib Resistance in Clear Cell Renal Cell Carcinoma (ccRCC)

**DOI:** 10.3390/cancers13174401

**Published:** 2021-08-31

**Authors:** María Armesto, Maitane Marquez, María Arestin, Peio Errarte, Ane Rubio, Lorea Manterola, Jose I. López, Charles H. Lawrie

**Affiliations:** 1 Molecular Oncology Group, Biodonostia Research Institute, 20014 San Sebastián, Spain; maria.armesto@biodonostia.org (M.A.); maitane.marquez@biodonostia.org (M.M.); maria.arestin@biodonostia.org (M.A.); ane.rubio2@gmail.com (A.R.); lmcareaga@gmail.com (L.M.); 2 Onena Medicines S.L., 20014 San Sebastián, Spain; Peio@onenameds.com; 3 Department of Pathology, Cruces University Hospital, 48903 Barakaldo, Spain; joseignacio.lopez@osakidetza.eus; 4 Biomarkers in Cancer Unit, Biocruces Research Institute, 48903 Barakaldo, Spain; 5 IKERBASQUE, Basque Foundation for Science, 48011 Bilbao, Spain; 6 Radcliffe Department of Medicine, University of Oxford, Oxford OX3 9BQ, UK

**Keywords:** renal cancer, sunitinib, resistance, miRNA, transcriptome, pathway analysis, clear cell renal cell carcinoma

## Abstract

**Simple Summary:**

Clear cell renal cell carcinoma (ccRCC) is a frequent cancer that causes more than 100,000 deaths every year. Treatment with drugs that target enzymes that help tumours grow such as sunitinib have greatly improved the prospects for ccRCC patients, however a large proportion of patients become resistant. We created sunitinib resistant cell lines and identified consequent changes in gene (and miRNA) expression by microarray analyses. Using this approach, we identified different pathways of resistance suggesting that tumour cells have many ways to overcome sunitinib treatment. We were able to overcome resistance in cells by inhibiting a protein, PD-L1, that is targeted by many immunotherapeutics currently in use for ccRCC patients suggesting a combination of immunotherapy and sunitinib may benefit patients. In addition, we identified miRNAs that are common to multiple resistance mechanisms suggesting they may be useful targets for future studies.

**Abstract:**

The anti-angiogenic therapy sunitinib remains the standard first-line treatment for meta static clear cell renal cell carcinoma (ccRCC). However, acquired resistance develops in nearly all responsive patients and represents a major source of treatment failure. We used an integrated miRNA and mRNA transcriptomic approach to identify miRNA:target gene interactions involved in sunitinib resistance. Through the generation of stably resistant clones in three ccRCC cell lines (786-O, A498 and Caki-1), we identified non-overlapping miRNA:target gene networks, suggesting divergent mechanisms of sunitinib resistance. Surprisingly, even though the genes involved in these networks were different, they shared targeting by multiple members of the *miR-17~92* cluster. In 786-O cells, targeted genes were related to hypoxia/angiogenic pathways, whereas, in Caki-1 cells, they were related to inflammatory/proliferation pathways. The immunotherapy target PD-L1 was consistently up-regulated in resistant cells, and we demonstrated that the silencing of this gene resulted in an increase in sensitivity to sunitinib treatment only in 786-O-resistant cells, suggesting that some ccRCC patients might benefit from combination therapy with PD-L1 checkpoint inhibitors. In summary, we demonstrate that, although there are clearly divergent mechanisms of sunitinib resistance in ccRCC subtypes, the commonality of miRNAs in multiple pathways could be targeted to overcome sunitinib resistance.

## 1. Introduction

Renal carcinomas are one of the most common types of cancer in the Western world, accounting for ~3% of adult tumours or more than 200,000 new cases each year [1]. Clear cell renal cell carcinoma (ccRCC) represents 80–90% of renal carcinomas and accounts for more than 100,000 deaths worldwide each year [2,3,4,5]. Nearly a third of patients present with locally advanced and/or metastatic disease that typically shows limited responsiveness to traditional therapies such as chemotherapy, radiotherapy, or cytokine therapy [6]. Moreover, ccRCC is a highly vascularized cancer that is frequently associated with mutations in the von Hippel–Lindau (VHL) gene that promotes the angiogenic pathway and can be further subclassified into those with proangiogenic and proinflammatory tumours [7].

The antiangiogenic therapy sunitinib (Sutent™) is currently the standard first-line treatment for metastatic ccRCC (mccRCC) [8,9]. Sunitinib is a small molecule inhibitor of multiple receptor tyrosine kinases (RTKs), including vascular endothelial growth factor receptor (VEFGR), platelet-derived growth factor receptors (PDGFR), fms-related tyrosine kinase 3 (FLT3), stem cell growth factor receptor KIT, and RET [10,11]. However, despite the clear improvements for ccRCC patients receiving this treatment, the clinical benefit of sunitinib on progression-free-survival (PFS) is limited, as more than half of patients do not respond to initial therapy, and of those that do, nearly all develop resistance after ~24 months [12,13]. Therefore, there is an urgent need for a better understanding of the molecular basis of sunitinib resistance in order to identify biomarkers of resistance that will allow for the detection of nonresponsive ccRCC patients that could benefit from up-front alternative treatment regimens, as well as developing new tools that could improve the treatment response in responsive patients.

**Figure 1:**
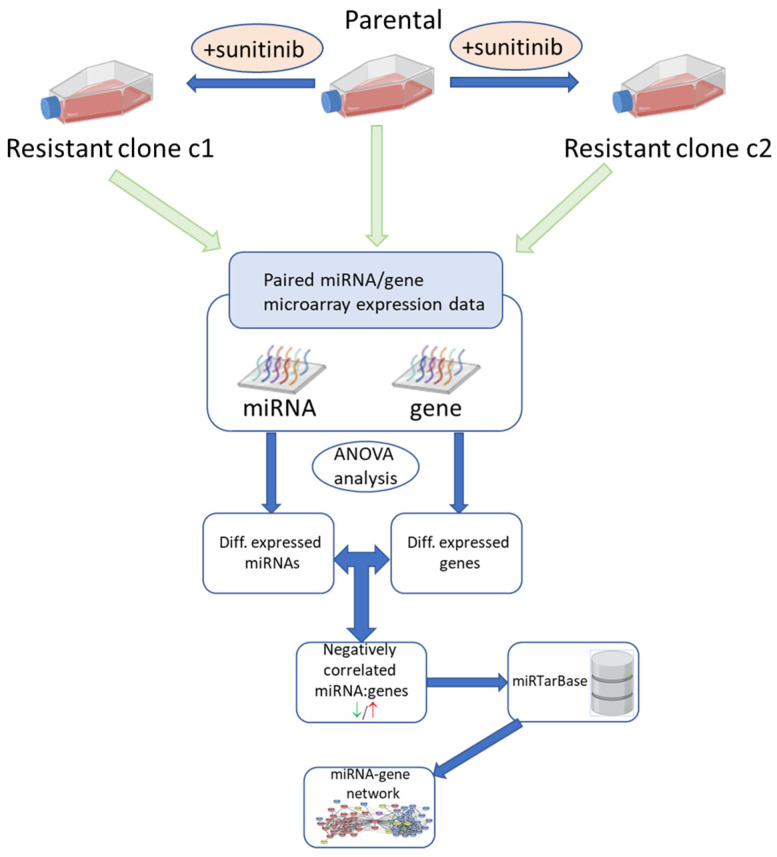
Schematic diagram of experimental workflow for the identification of miRNA:gene interactions involved in sunitinib resistance.

Although many publications have investigated the molecular basis of sunitinib resistance [14,15,16,17,18,19], and several have considered the role of microRNAs (miRNAs) [20,21,22,23,24,25,26,27], only a few have taken an integrated genomic approach to identify miRNA-target gene interactions that can give functional insights into resistance mechanisms [28,29]. Therefore, we used generated multiple sunitinib-resistant clones in primary tumour ccRCC cell lines that are VHL-defective (786-O and A498) and the metastatic, VHL-functional, Caki-1 cell line. Changes in the expression of both miRNAs and genes were elucidated in the resistant clones by microarray analysis and differentially expressed genes that were targeted by differentially expressed miRNAs were identified by network analysis (Figure 1). These results were confirmed by both mRNA and protein levels and the immunotherapy target PD-L1 was identified as being up-regulated in resistant cell lines. Silencing of PD-L1 was demonstrated to restore the sensitivity of resistant 786-O cells.

## 2. Results

### 2.1. Generating In Vitro Models of Sunitinib Resistance

Sunitinib-resistant 786-O, A498, and Caki-1 clones were generated by serial passage in increasing concentrations of sunitinib until 10 µM was reached. Two independent resistant clones were developed for each cell line and the IC_50_ of each clone was calculated by MTT assay (Figures S1–S3 and Table 1).

As can be seen from Table 1, the average IC_50_ value of the sunitinib-resistant clones c1 and c2 were significantly higher than their respective parental control cell lines.

### 2.2. Non-Coding RNA and Gene Expression in Sunitinib-Resistant Cells

**Figure 2:**
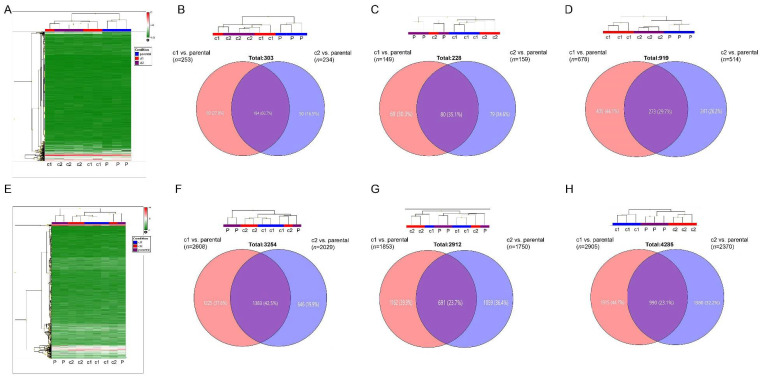
Microarray analysis of gene and miRNA expression in resistant and parental control cell lines: (**A**) Representative unsupervised cluster analysis of miRNA expression (786-O cells). (**B**–**D**) (Top) Dendrogram of unsupervised cluster analysis showing relationship between samples at level of miRNA expression; (Bottom) Venn diagram depicting differentially expressed miRNAs in resistant clones relative to parental control cells. (**B**) 786-O cells, (**C**) A498 cells, (**D**) Caki-1 cells. (**E**) Representative unsupervised cluster analysis of gene expression (786-O cells). (**F**–**H**) (Top) Dendrogram of unsupervised cluster analysis showing relationship between samples at level of gene expression; (Bottom) Venn diagram depicting differentially expressed genes in resistant clones relative to parental control cells. (**F**) 786-O cells, (**G**) A498 cells, (**H**) Caki-1 cells. The original heatmap analyses are shown in Figure S4. Differentially expressed miRNAs and genes that are common between the resistant clones are listed in (Tables S1–S6).

In order to look at which miRNAs and genes were involved in the resistant phenotype of the cell lines, we carried out microarray analyses. Unsupervised cluster analysis demonstrated that the miRNA expression profile of the resistant cell lines differed from that of the parental control cell lines in all three cell lines (Figure 2A–D). Moreover, there was a clear difference in miRNA expression between c1 and c2 in cell lines, suggesting different mechanisms of resistance, although this difference was most pronounced in Caki-1 cells.

Using ANOVA analysis, we identified 253 differentially expressed miRNAs between resistant 786-O clone c1 and the parental control, of which 184 were up-regulated and 69 were down-regulated. There were 234 miRNAs differentially expressed miRNAs between clone c2 and parental control; 182 were up-regulated and 52 were down-regulated. Over 60% (184/303) of each of these differentially expressed miRNAs were common to both clone c1 and clone c2 (Figure 2A,B; Table S1). In A498 cells, we identified 102 differentially expressed miRNAs between clone c1 and the parental control, of which 68 were up-regulated and 34 were down-regulated. For clone c2, 107 miRNAs were differentially expressed when compared with the parental; 61 were up-regulated and 46 were down-regulated. Nearly 32% (51/158) of these miRNAs were commonly dysregulated in the two clones (Figure 2C; Table S2). In the Caki-1 cell line, we identified 678 differentially expressed miRNAs between clone c1 and the parental control, of which 324 were up-regulated and 354 were down-regulated. For the clone c2, 514 miRNAs were differentially expressed when compared with the parental; 245 were upregulated and 269 were downregulated. Nearly 30% (273/919) of these miRNAs were commonly dysregulated in the two clones (Figure 2D; Table S3).

In addition to miRNA analyses, we carried out gene expression analysis on the same samples using Affymetrix Clariom D microarrays. Unsupervised cluster analysis of gene probes (intensity > 50) showed a similar relationship between samples as with the miRNAs (Figure 2E–H). In other words, there was a distinct gene profile between the parental control cell lines and the resistant cell lines, and the two resistant clones, c1 and c2, had distinct gene expression profiles. Similar to miRNA expression, these differences were most pronounced in Caki-1 cells. There were 4869 gene probes identified as being differentially expressed (*p* < 0.05; >2 or <−2-fold) between 786-O c1 and parental cells, 2608 of which encoded for annotated genes (1913 up-regulated and 695 down-regulated). In clone c2, there were 3994 differentially expressed genes, of which 2029 encoded for annotated genes (1397 up-regulated and 632 down-regulated). There were 1383 genes in common (43% of 3254) (Figure 2E,F; Table S4). For A498 cells, 3019 probes were identified as being differentially expressed between c1 and parental cells, of which 1523 encoded for annotated genes; 972 of these were up-regulated and 551 were down-regulated. There were 2953 probes differentially expressed between c2 and parental cells, of which 1411 encoded genes were comprised of 806 up-regulated and 605 down-regulated genes. There were 621 of 2313 (27%) genes that were commonly dysregulated in both clones (Figure 2G; Table S5). In the Caki-1 cell line, there were 7059 genes differentially expressed between c1 and parental cells, of which 2905 were annotated (883 up-regulated, 2022 down-regulated). For clone c2, 6201 genes were differentially expressed, 2370 of which were annotated (1153 up-regulated, 1217 down-regulated). A total of 990 genes (23%) were common between c1 and c2 (Figure 2H; Table S6).

### 2.3. Interaction Network Analysis between Differentially Expressed Genes and miRNAs

In order to identify which of the differentially expressed genes were regulated by miRNAs, we mapped the differentially expressed miRNA and gene expression data sets for each resistant clone to a network containing experimentally validated miRNA-gene target interactions (*n* = 3502) that were obtained from the miRTarBase database [30]. For the 786-O c1 cells, 545 (19%) of differentially expressed genes and miRNAs mapped to this network from 253 and 2608 differentially expressed miRNAs and genes, respectively (*n* = 2861), and 453 (20%) from the 234 and 2029 differentially expressed miRNA and genes from c2 (*n* = 2263). In the A498 cell line, out of the 1625 differentially expressed genes and miRNAs for c1 (102 miRNAs and 1523 genes), 338 (21%) mapped to the miRNA:gene interaction network, whereas, for c2, 313 (21%) of the 1518 genes and miRNAs (*n* = 107 and 1411, respectively) were present in the network. For Caki-1, out of the 3583 differentially expressed genes and miRNAs in c1 (*n* = 678 and 2905, respectively), 696 (19%) were mapped to the interaction network, and in c2 there were 577 (20%) of 2884 (514 and 2370 miRNAs and genes, respectively).

**Figure 3:**
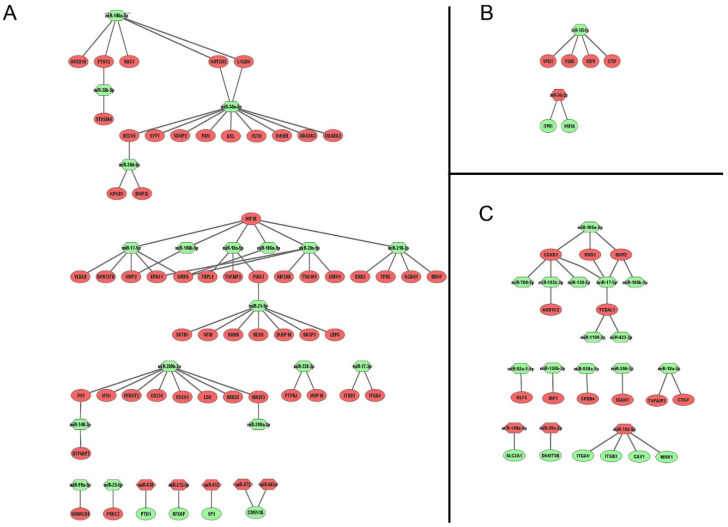
miRNA-gene network analysis in (**A**) 786-O, (**B**) A498, and (**C**) Caki-1 cells. Ellipsoidal nodes represent target genes and hexagonal nodes miRNAs, red colour denotes colour denotes upregulation and green downregulation.

The mapped differentially expressed miRNAs and genes were separated into miRNAs that were up-regulated and genes that were down-regulated and vice versa. These lists were used to create networks from miRNA:genes that were common to both c1 and c2 clones (Figure 1 and Figure 3). For 786-O cells, for example, there were 76 gene:miRNA interactions, 71 interactions involving 18 down-regulated miRNAs with 53 different genes, and 5 interactions with 5 up-regulated miRNAs with four different genes (Figure 3A; Table 2). For A498, there was only one commonly up-regulated miRNA (*miR-34c-5p*) that targeted two genes, and one down-regulated miRNA (*miR-145-5p*) that targeted four genes—a total of six miRNA:gene interactions (Figure 3B; Table 2). For the Caki-1 cell line, there were 26 miRNA:gene interactions, three up-regulated miRNA targeting six genes, and 12 down-regulated miRNAs targeting twelve different genes (Figure 3C; Table 2). Only two genes (*ITGB3* and *TNFAIP3*) were in common between the cell lines (i.e., 786-O and Caki-1).

**Figure 4:**
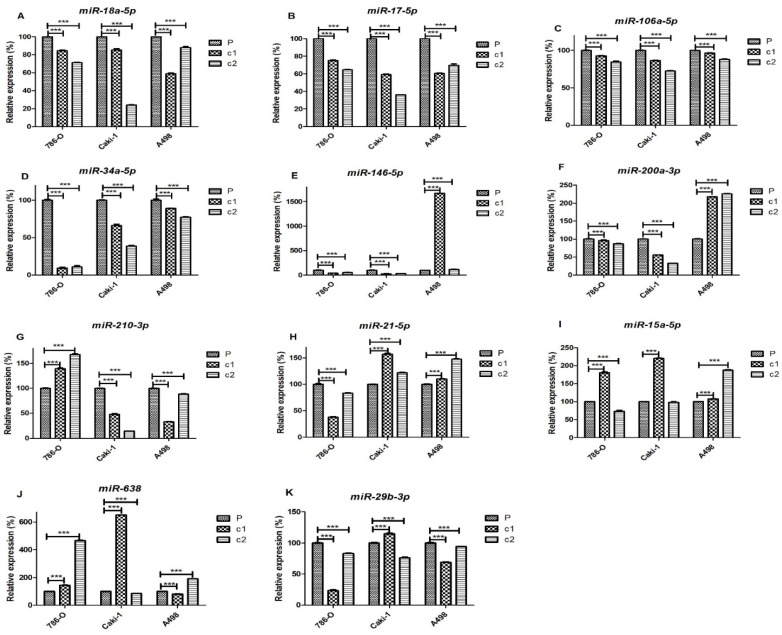
miRNA expression in resistant clones and parental samples of 786-O, Caki-1, and A498 cell lines: The levels of (**A**) *miR-18a-5p*, (**B**) *miR-17-5p*, (**C**) *miR-106-5p*, (**D**) *miR-34a-5p*, (**E**) *miR-146-5p*, (**F**) *miR-200a-3p*, (**G**) *miR-210-3p*, (**H**) *miR-21-5p*, (**I**) *miR-15a-5p* (**J**) *miR-638*, and (**K**) *miR-29b-3p* in both clones and parental cells were measured by qRT-PCR using *snoRNU48* as a reference gene. Expression is shown relative to parental expression. Experiments were performed in biological and technical triplicate. The significance of comparisons are denoted by *** *p* < 0.001.

On the basis of their role in the sunitinib-resistance miRNA-gene interaction networks, we selected eleven miRNAs and seven genes for further analysis by qRT-PCR. miRNAs *miR-18a-5p, miR-17-5p, miR-106a-5p, miR-34a-5p, miR-146-5p, miR-200a-3p, miR-210-3p, miR-21-5p, miR-15a-5p, miR-638*, and *miR-29b-3p* were measured in the cell lines by qRT-PCR (Figure 4A–K respectively). As can be seen from these results, levels of multiple members of the *miR-17~92* cluster (i.e., *miR-18-5p, miR- miR-17-5p*, and *miR-106-5p* (Figure 4A–C respectively)) were significantly down-regulated in all of the resistant clones relative to the parental cell lines in 786-O, A498, and Caki-1 cells. Similarly, we observed significant down-regulation of *miR-34a-5p* in resistant clones of all the three cell lines (Figure 4D). *miR-146-5p* was down-regulated in both clones of 786-O and Caki-1, and clone c2 of A498 was up-regulated more than 15-fold in c1 of A498 cells (Figure 4E). *miR-200a-3p* was significantly down-regulated in both 786-O and Caki-1 cells but not A498 cells where this miRNA was up-regulated in resistant cells (Figure 4F). Caki-1 and A498 cells showed significant down-regulation of *miR-210-3p* in resistant clones, whereas this miRNA was up-regulated in 786-O cells (Figure 4G). *miR-21-5p* was up-regulated in A498 and Caki-1 cells but down-regulated in 786-O cells (Figure 4H). In contrast to the aforementioned miRNAs, *miR-15a-5p, miR-638*, and *miR-29b-3p* differed in expression between different resistant clones. For example, *miR-15a-5p* was up-regulated in c1 but not c2 in both 786-O and Caki-1 cells but up-regulated in both A498 clones (Figure 4I). For *miR-638*, both resistant clones of 786-O cells, as well as c1 of Caki-1 and c2 of A498 cells, were up-regulated compared to parental cells, whereas c2 of Caki-1 and c1 of A498 cells were down-regulated (Figure 4J). *miR-29b-3p* was down-regulated in both clones of 786-O and A498, but only c2 of Caki-1 cells while c1 was up-regulated compared to the parental Caki-1 cells (Figure 4K).

**Figure 5:**
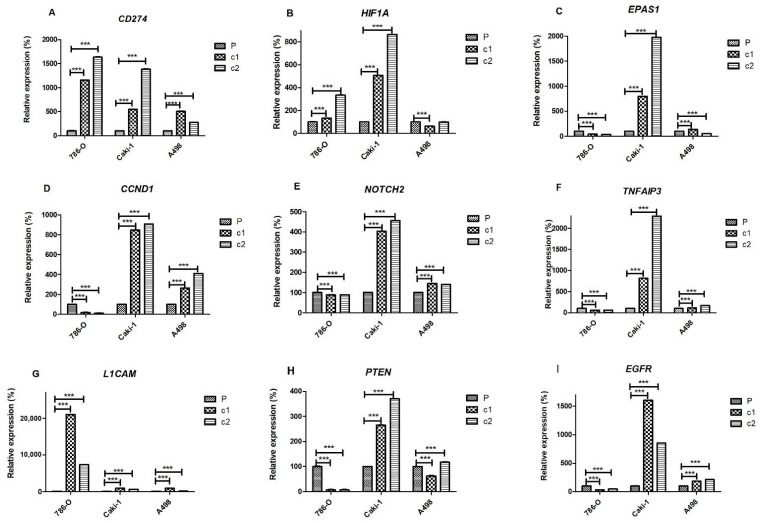
Gene expression in resistant clones and parental cells of 786-O, Caki-1, and A498 cell lines. Levels of (**A**) *CD274*, (**B**) *HIF1A*, (**C**) *EPAS1*, (**D**) *CCND1*, (**E**) *NOTCH2*, (**F**) *TNFAIP3*, (**G**) *L1CAM*, (**H**) *PTEN*, and (**I**) *EGFR* genes measured by qRT-PCR using *GAPDH* as the reference gene. Expression levels in resistant clones are depicted relative to their respective parental cells. All experiments were performed in biological and technical replicates. The significance of comparisons is denoted by *** *p* < 0.001.

Of the nine genes that were measured by qRT-PCR, only *CD274* (encoding for PD-L1 protein) displayed a consistent expression pattern (i.e., up-regulated) in all three cell lines with both resistant clones (Figure 5A). *HIF1A* was up-regulated in both c1 and c2 of 786-O and Caki-1 cells, as well as c2 of A498, but down-regulated in c1 of A498 (Figure 5B). The closely related gene *EPAS1* (encoding for HIF2α protein) was also up-regulated in both clones of Caki-1 (and c1 of A498), but down-regulated in 786-O-resistant clones and c2 of A498 cells (Figure 5C). A similar pattern was observed for *CCND1, NOTCH2*, and *TNFAIP3,* which were also down-regulated in resistant clones of 786-O cells but up-regulated in both Caki-1 and A498-resistant clones (Figure 5D, 5E and 5F, respectively). In contrast, levels of *LICAM* were up-regulated in both resistant clones of 786-O and Caki-1 cells but only up-regulated in c1 of A498 cells (Figure 5G). Levels of *PTEN* were down-regulated in 786-O cells and c1 of A498 cells but up-regulated in Caki-1 cells and c2 of A498 cells (Figure 5H). Levels of *EGFR* were similarly down-regulated in 786-O and A498-resistant clones but down-regulated in 786-O-resistant clones (Figure 5I).

**Figure 6:**
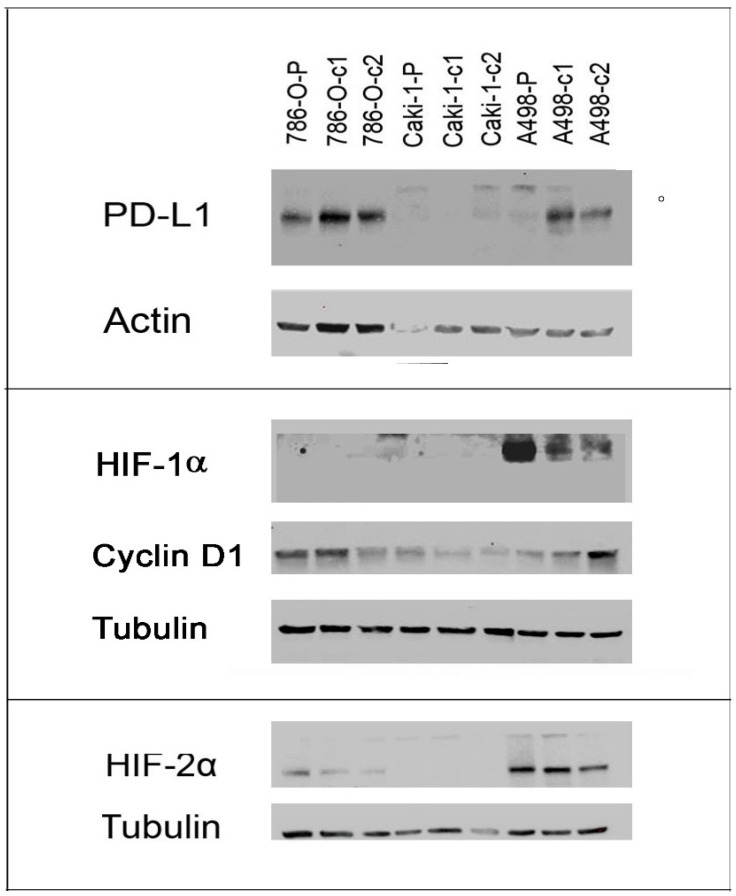
Protein expression in resistant clones and parental control cells of 786-O, Caki-1, and A498 cell lines.

We tested the protein levels of PD-L1 (*CD274*), HIF1α (*HIF1A*), HIF2α (*EPAS1*), and cyclin D1 (*CCND1*) by Western blot analysis (Figure 6; Table 3). These results were largely consistent with the qRT-PCR results. For example, there was a clear increase in PD-L1 expression in the resistant clones of both 786-O and A498 cells compared to the parental cells, and a decrease in levels of HIF2α. For both proteins, however, we observed no expression in Caki-1 cells. Moreover, we observed no expression of HIF1α in Caki-1 cells or 786-O cells, despite repeated replicates. In contrast, in A498 cells, HIF1α was expressed and downregulated in resistant clones. *CCND1* was down-regulated in 786-O-resistant clones but up-regulated in resistant clones of A498 cells. Although expressed in Caki-1 cells, cyclin D1 protein appears to be down-regulated in contrast to the up-regulation of mRNA levels observed by qRT-PCR.

### 2.4. Gene Ontology and Pathway Analyses

In order to further investigate the potential role of miRNA-regulated genes in sunitinib resistance, we carried out ontology analysis by KEGG pathway enrichment analysis (Tables S7–S9). The number of significantly enriched pathways was highest (*n* = 12) in 786-O cells and lowest in A498 cells (*n* = 6), reflecting the different numbers of genes associated with miRNAs in these cell lines (Figure 3). Reassuringly, the most significant pathway in this analysis for 786-O cells was *miRNAs in cancer* (*p*-value 4 × 10^−9^), which was also significantly enriched in Caki-1 cells despite having non-overlapping genes. The second most highly enriched pathway in this analysis was *proteoglycans in cancer* (*p*-value 1.6 × 10^−5^), which was also significantly enriched in A498 (*p*-value 8.7 × 10^−3^) and Caki-1 cells (*p*-value 2.2 × 10^−5^); again, these pathways had non-overlapping genes in the three cell lines. Other significant pathways commonly shared between different cell lines were the *Human papillomavirus infection* in 786-O and Caki-1 cells (*p*-values 5.5 × 10^−4^ and 1.4 × 10^−3^ respectively) and *fluid shear stress* pathways (*p*-values 7.7 × 10^−3^ and 1.4 × 10^−3^, respectively). The *PI3K-Akt signalling pathway* was also common between A498 and Caki-1 cells, but not 786-O (*p*-values 7.7 × 10^−3^ and 1.4 × 10^−3^, respectively).

### 2.5. Silencing of PD-L1 in 786-O Sunitinib-Resistant Cells Results in an Increased Sensitivity to Sunitinib

**Figure 7:**
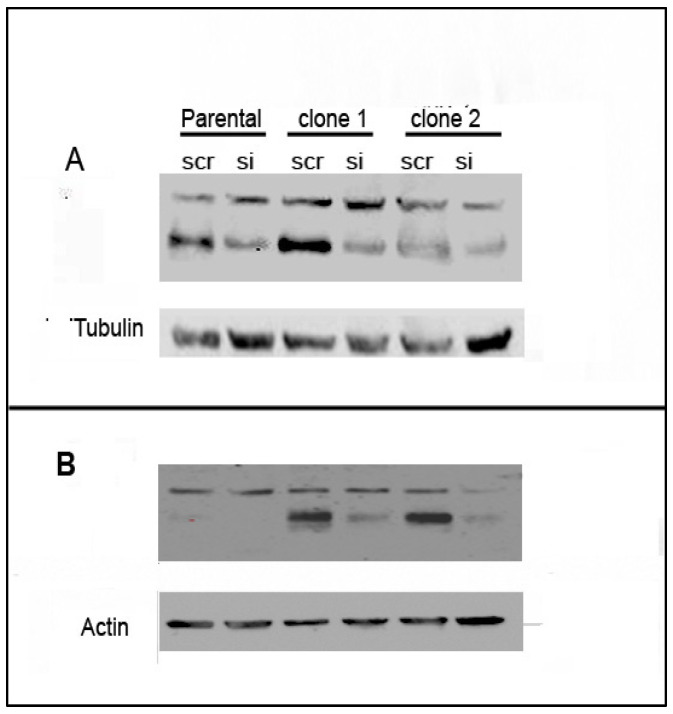
PD-L1 protein expression in 786-O (**A**) and A498 (**B**) parental and resistant cells after treatment with siRNA against CD274 (or scramble control).

As we observed a consistent up-regulation of *CD274* (PD-L1) in all of the resistant clones of all three cell lines and increased protein expression, in 786-O and A498 cells, at least, we hypothesised that this molecule would play an important role in the resistant phenotypes. We therefore silenced this gene in resistant and parental cells to investigate the effect on the resistant phenotype. After confirming the silencing by qRT-PCR (Figure S4) and protein level (Figure 7; Table 4), we carried out sunitinib dose experiments by MTT assay (Table 5). We observed that the silencing of *CD274* led to a significant increase in the sensitivity of 786-O-resistant clones but not in parental cells treated with the same siRNA. This effect was more pronounced in c1 cells, which is consistent with the increased silencing in this clone compared to c2 (84% and 72% reduction in c1 at and 38% and 72% in c2 at 48 h and 72 h, respectively: Figure S5). In contrast, the silencing of *CD274* in A498 or Caki-1 cells did not increase the sensitivity of resistant clones (or parental cells) to sunitinib treatment.

## 3. Discussion

Inactivation of the *VHL* gene and activation of the HIF-VEGF pathway are the major molecular hallmarks of renal carcinoma and form the basis of antiangiogenic therapy such as sunitinib. Sunitinib remains the first-line treatment for mccRCC, and acquired resistance and tumour metastases are the main causes of treatment failure [31]. Consequently, several mechanisms have been proposed, including the up-regulation of *FGF1* [32], the induction of epithelial to mesenchymal transition (EMT) and alternative growth factor signaling [33], and the down-regulation of *PTEN* [18], amongst others. In addition to genes, several studies have established the role of miRNAs and other non-coding RNAs (ncRNAs) in sunitinib resistance [21,23,26,34]. However, very few have used an integrated genomic approach to identify target genes regulated by miRNAs—an approach that lends itself to the possibility of using miRNA-based therapeutics to overcome sunitinib resistance in ccRCC patients.

We, therefore, developed an in vitro model of sunitinib resistance in three different cell lines (786-O, A498, and Caki-1) through prolonged exposure to the drug. All these cell lines were originally sensitive to sunitinib, with an IC_50_ value less than 10 µM (average 8.8 µM), the concentration reached in patient tumour tissue [33]. The generated resistant clones had a significantly higher amount of IC_50_ values greater than 10 µM (average 15.6 µM). Although several studies have investigated miRNA expression in response to sunitinib-treatment, the vast majority have looked at expression after a single dose [22,24,25], rather than prolonged exposure, which could be argued to more accurately reflect acquired resistance [21,23,35].

Unsupervised cluster analysis of miRNA and gene expression data showed that resistant cells not only differed from sunitinib-sensitive cells, but also differed between resistant clones, suggesting differing mechanisms of resistance. This was confirmed by the low levels of overlap (<50%) of differentially expressed genes and miRNAs between the clones. There were 3254, 2313, and 4285 differentially expressed genes identified in 786-O, A498, and Caki-1 cells, respectively. However, gene enrichment analysis using the KEGG pathway database failed to detect any significantly enriched pathways amongst these gene datasets, suggesting that many of these genes were only indirectly linked to the resistant phenotype. In order to resolve this issue, and bearing in mind the association of miRNAs with sunitinib resistance [23,25,26,27,36,37,38], we used an integrated genomic approach to identify miRNA-regulated target genes by molecular interaction network analyses. Using this methodology, we identified 76, 6, and 26 miRNA:gene interactions consistently involved in sunitinib resistance for 786-O, A498, and Caki-1 cells, respectively. The much larger number of interactions in 786-O cells most likely reflects the much higher degree of overlap in genes and miRNAs found in this cell line between the clones c1 and c2 (60% cf 32% (A498) and 30% (Caki-1) for miRNAs and 43% vs. 27% vs. 20% of genes).

When we repeated the gene enrichment analysis on the miRNA-regulated genes, we a observed significant enrichment for the ‘*proteoglycans in cancer*´ KEGG pathway in all three cell lines, even though the corresponding gene lists were non-overlapping. Other shared pathways were ‘*human papillomavirus infection*’, ‘*fluid shear stress*’, and ‘*PI3K-Akt signalling*’ pathways. Consistent with these findings, Chen et al. also reported that ‘*proteoglycans in cancer*’ and ‘*PI3K-Akt signalling*’ pathways were amongst the most significant pathways identified by meta-analysis of 88 gene expression and next generation sequencing data sets from sunitinib resistance studies containing both in vitro and inpatient-derived xenograft models [29]. Yamagouchi et al. likewise identified the *PI3K-AKT pathway* as significant by KEGG analysis of sunitinib-resistant cells [23]. Moreover, the genes identified in these studies were non-overlapping, with those identified in our study implying a functional relevance of these pathways in sunitinib resistance. Proteoglycans are major components of the extracellular matrix and play important roles in many facets of cancer, including proliferation, adhesion, angiogenesis, and metastasis [39]. Recently, Rausch et al. described the morphometric changes that occur in sunitinib-resistant clones, in which the authors linked the changes of more than 70 genes to cell adhesion, including many proteoglycans [35].

We observed that multiple members of the *miR-17~92* clusters (i.e., *miR-17-5p, miR-17-3p, miR-18a-5p, miR-18a-3p, miR-20a-5p*) and paralogue clusters, including all the members of the *miR-106b~miR-25* (*miR-106b, miR-93-5p,* and *miR-25-5p*) cluster and the *miR-106a-5p,* which is encoded by the *miR-106a~363* cluster, were down-regulated in resistant ccRCC clones. The down-regulation of *miR-18a-5p*, at least, has previously been reported in other sunitinib-resistant ccRCC cell lines (ACHN and RCC23) [23]. Intriguingly, network analyses revealed that the target genes of these miRNAs were non-overlapping in the different cell lines (Table 2). This suggests the involvement of differing gene pathways in resistance mechanisms but implies a convergent regulatory role of these miRNAs in ccRCC, making them potential common targets for modulation that surely warrants further investigation and could potentially be targeted to overcome sunitinib resistance. For example, in 786-O cells, target genes were generally hypoxia and angiogenic-related (i.e., *EPAS1, HIF1A, MMP2,* and *VLDLR*), whereas, in Caki-1 cells, target genes (i.e., *CCND1, BMP2, TCEAL1*, and *RND3*) were involved in inflammatory, proliferation, and migration, possibly a reflection of the metastatic phenotype of these cells. Indeed it is tempting to infer that these mechanisms reflect the subclassification of ccRCC into angiogenic and inflammatory tumours that has recently been proposed by Brugarolas et al. [7].

The most regulated gene in 786-O cells was *HIF1A,* which was regulated by down-regulation of multiple members of the *miR-17~92* cluster and *miR-210*, highly suggestive of playing a major role in sunitinib resistance in this cell line. The role of HIF1α in sunitinib treatment and resistance has long been recognised [40]. Yamagouchi et al. similarly found that *HIF1A* was up-regulated in sunitinib-resistant cell lines and that it was targeted by *miR-18a-5p* [23]. Even though there was a clear increase in *HIF1A* expression in the resistant clones of 786-O and Caki-1 cells (Figure 5; Table 3), the HIF1α protein was not detected in these cell lines (Figure 6; Table 4). This is consistent with previous studies, that have shown a lack of protein expression in 786-O cells due to mutations [41,42], and even though Caki-1 cells do encode the intact *HIF1A* gene, the protein is only expressed under hypoxic conditions [43]. The fact that the *HIF1A* transcript, but not the protein, is induced in these cell lines could have functional significance in the resistance mechanism, as several long non-coding RNAs (lncRNAs) are encoded within this gene [44,45], and this is an area we are currently investigating. Although A498 cells also contain a mutated *HIF1A* gene, the HIF1α protein is expressed constitutively under normoxic conditions due to defective *VHL* [43]. We observed that HIF1α expression was down-regulated in A498-resistant clones, a characteristic that was previously described to be the result of sunitinib-associated proteosome degradation [41].

In the Caki-1 miRNA:target gene network, *CCND1* was the most regulated gene which was potentially targeted by five out of thirteen (38%) of down-regulated miRNAs, none of which were found in the 786-O network. Indeed, in contrast to Caki-1 (and A498) resistant clones, 786-O-resistant clones were characterised by *CCND1* mRNA and cyclin D1 protein down-regulation. *CCND1* is not only a marker of proliferation and tumour growth [46], but is also associated with metastatic potential [46,47]. Although the down-regulation of members of the *miR-17~92* cluster is consistent with the up-regulation of *CCND1* observed in Caki-1 and A498-resistant clones [48], the same miRNAs are also down-regulated in 786-O-resistant clones, suggesting a different regulatory mechanism for this cell line, perhaps through the direct targeting by HIF2α [49], which is down-regulated at mRNA and protein levels. Intriguingly, although *CCND1* was strongly up-regulated (8–10-fold) in resistant clones of Caki-1 cells, cyclin D1 protein was down-regulated, as has recently been described [35], suggesting post-transcriptional regulation.

The PD-L1 gene (*CD274*) was also identified in our network analysis as being targeted by *miR-200a*, which we had previously demonstrated was characteristically down-regulated in ccRCC [50], and that was down-regulated in both 786-O and Caki-1-resistant clones (but not A498 cells). *miR-200* has been shown to directly target *CD274*/PD-L1 expression, and, in concert with ZEB1, to play an important role in the initiation of metastasis via the induction of epithelial-to-mesenchymal transition (EMT) and CD8+ TIL immunosuppression [51]. In addition to *miR-200, miR-34a* has also been identified as an important regulator of PD-L1 expression [52]. Indeed, a phase 1 clinical trial, using a liposomal mimic of this miRNA (MRX34), included renal carcinoma patients, although the trial was halted due to serious adverse effects [53]. We observed that the levels of *miR-34a* were significantly down-regulated in the resistant clones of all three cell lines. An increase in PD-L1 protein expression in response to transient sunitinib treatment has previously been reported in 786-O and A498 cell lines [17,54]. We extended these observations to the resistant clones of these cell lines, as well as to *CD274* mRNA expression. Indeed, this was the only gene that we found to be consistently up-regulated in all three of the cell lines in this study. It should be noted, however, that we were unable to detect PD-L1 protein expression in the Caki-1 cell line, an observation consistent with previous studies [55,56], presumably due to the lack of HIF1α expression under normoxic conditions in this cell line that also regulates PD-L1 expression [54].

To explore the role of PD-L1 further in the resistant phenotype, we silenced this gene in the three cell lines and observed an increase in the sensitivity to sunitinib of the 786-O-resistant clones but not the parental counterpart cells, nor the Caki-1 or A498 cells. The difference between the response between the cell lines suggests again that there are different resistance mechanisms operating between the cell lines, and that the PD-L1-associated mechanism is most important in 786-O cells. In contrast, in Caki-1 cells, where PD-L1 is not expressed, resistance appears to be regulated through cyclin D1, although this remains to be experimentally confirmed. The lack of effect of PD-L1 silencing on A498 cells, however, is somewhat more surprising, suggesting that the down-regulation of HIF1α that we observed in resistant clones is probably a more dominant mechanism for resistance than PD-L1, as it has been demonstrated that ectopic expression of HIF1α in 786-O cells make them more susceptible to sunitinib [41]. These results suggest that ccRCC patients with *VHL* gene mutations (>50% of patients) [57] that do not express HIF1α (~70% of ccRCC patients [58]) could have an improved response to sunitinib treatment through targeting of PD-L1 by checkpoint inhibitor antibodies such as avelumab that already has FDA-approval for combination treatment in ccRCC [59]. Consistent with this hypothesis, Guo et al. demonstrated that a combination of anti-PD-L1 and sunitinib significantly reduced tumour progression in vivo [17]. Indeed, although immunotherapy targeting the PD-1/PD-L1 axis shows great promise for ccRCC, only 15–25% of patients respond when given it as a monotherapy [60], and there is increasing movement towards combination therapy of antiangiogenic agents and immunotherapy [59,61,62,63,64]. We are not aware, however, of any trials to date that have combined sunitinib with anti-PD-L1 immunotherapy. We recognise however, that cell lines may not give a complete reflection of what occurs in ccRCC patients.

In summary, the present study has demonstrated that the use of in vitro models of sunitinib resistance, combined with an integrated genomic approach, can identify divergent mechanisms of sunitinib resistance that could be exploited for the benefit of ccRCC patients.

## 4. Materials and Methods

### 4.1. Generation of Sunitinib-Resistant ccRCC Cell Lines

The ccRCC cell lines 786-O (ATCC^®^ CRL1932™), A498 (ATCC^®^ HTB 44™), and Caki-1 (Caki-1ATCC^®^ HTB46™) were obtained from the American Type Culture Collection (ATCC, Manassas, VA, USA). 786-O and A498 cells were grown in RPMI and MEM media respectively, in the presence of 10% fetal calf serum (FCS), 1% *L*-glutamine and 1% penicillin-streptavidin (Fisher Scientific, Waltham, MA, USA). Caki-1 cells were grown in McCoy’s 5A (modified) medium with 10% FCS + 1% *L*-glutamine and 1% penicillin-streptavidin (Fisher Scientific, Waltham, MA, USA). Two resistant clones for each cell line were generated by gradually exposing the cells to increasing concentrations of sunitinib (0.5 µM increase per passage) until a final concentration of 10 µM. For each increase in sunitinib concentration, cells were passaged at least twice to remove dead cells. Parental control cells were passaged in parallel without the addition of sunitinib.

### 4.2. Cell Proliferation Assay

Cells were seeded onto 96-well plates at a density of 2 × 10^3^ cells per well and allowed to attach for 24 h. Afterwards, the cells were treated with differing doses of sunitinib (i.e., 1.25, 2.5, 5, 7.5, 10, 15, 20, 30, and 40 µM) or DMSO as a negative control. Seventy-two hours later, MTT (3-(4,5-Dimethylthiazol-2-yl)-2,5-Diphenyltetrazolium Bromide) was added to the cells before a further incubation at 37 °C for three hours. The reaction was stopped by the addition of DMSO and the resulting absorbance at 570 nm was measured using a Halo LED 96 plate reader (Dynamica Ltd., Livingston, UK). Each sample was measured in triplicate wells and each experiment carried out a minimum of three times.

### 4.3. RNA Extraction and Microarray Analysis

Total RNA was extracted from cell line material using Trizol in accordance with the manufacturer’s instructions (Life Technologies, Paisley, UK). One µg of total RNA was used for Affymetrix Genechip miRNA v.4.0 microarrays, and 200 ng of DNAse treated total RNA were used for Clariom D human microarrays to measure miRNA and gene expression, respectively. The RNA was labelled and hybridised to microarrays in accordance with the manufacturer’s instructions (Affymetrix, CA, USA).

Resulting raw intensity data (i.e., cel files) were imported and analysed within Transcriptome Analysis Console (TAC) software version 4.0.2 (Affymetrix, CA, USA). Using this software, we identified differentially expressed miRNAs or genes on the basis of >2-fold up- or down-regulation along with Benjamini–Hochberg multiple corrected *p* values < 0.05. All microarray data was MIAME compliant, and raw data was deposited in the GEO database (GSE183140). For miRNA microarray analysis, probes were filtered for only human mature miRNAs (i.e., hsa-miR*) (* means wild-card i.e., any miR) and, for gene expression analysis coding, genes were classified as probes and filtered using the group variable ‘coding’ or ‘multiple complex’, before removing non-annotated genes that only had a numerical Aceview description.

### 4.4. Interaction Network Analysis

In order to identify differentially expressed genes that are likely to be regulated by miRNAs, we used the Cytoscape program (v3.8.2) (NIH, Bethesda, MD, USA) [65] to create an (experimentally validated) miRNA-target gene network. In brief, we imported and created a reference network of experimentally validated interactions (*n* = 10,755) from the miRTarBase dataset consisting of 3502 genes and miRNAs [30]. Differentially expressed miRNAs and genes from microarray analyses were imported into the program and mapped to the reference network. Mapped miRNA:target gene interactions were filtered according to inverse correlations (i.e., up-regulated miRNAs and down-regulated genes and vice versa) for each individual clone, and the intersection between the clonal networks was used to produce common networks, as depicted in Figure 3. These common networks were used for ontology analysis using the STRING app (version 1.6.0) (University of California, San Francisco, CA, USA) to interrogate the KEGG pathway database (release 95.2) implemented in Cytoscape. An overview of the workflow used is depicted in Figure 1.

### 4.5. Quantitative RT-PCR (qRT-PCR)

To measure levels of individual miRNAs by qRT-PCR, we used 200 ng of total RNA. The RNA was reverse transcribed using the Taqman Megaplex miRNA pool A according to the manufacturer’s instructions (Applied Biosystems, Warrington, UK), except in the case where specific miRNAs were not present in this pool, in which individual primers were used. qPCR was carried out with individual Taqman probes in triplicate using a LightCycler^®^ 96 System machine (Roche, Basel, Switzerland). The snoRNA *RNU48* was used as the reference gene for miRNA quantification as previously described [66], and *GAPDH* was used as a control for gene expression. In brief, the mean Ct value of each triplicate was quantified by the ΔC_t_ method (i.e ΔC_t_ = mean C_t_ of *RNU48/GAPDH* minus the mean C_t_ of miRNA/gene of interest). All qRT-PCR assays were carried out in technical and biological triplicate and expression levels were compared using the Mann–Whitney independent *t*-test (Graphpad Prism v.5.0, La Jolla, CA, USA)**.**

### 4.6. PDL-1 Silencing

Cells (4 × 10^4^) were transfected with either 5 nM of ON-TARGET plus human CD274 SMART pool siRNA or a non-targeting scramble control (Dharmacon, Lafayette, CO). Transfection was carried out in 12-well plates using DharmaFECT^TM^ reagent (Dharmacon) according to the manufacturer’s protocol. Cells were harvested at 48-, 72-, and 96-h post-transfection, and RNA was extracted using Trizol (Fisher Scientific).

### 4.7. Western Blotting

Cells were washed with ice-cold PBS twice before lysis in RIPA buffer containing Halt^TM^ protease and phosphatase inhibitor cocktail (Thermo Scientific, Waltham, MA, USA). Protein concentrations were measured by BCA protein assay (Thermo Scientific, Waltham, MA, USA), and equal amounts of protein were run on 10% Mini-PROTEAN TGX Precast Protein Gels (Bio-Rad, Hercules, CA, USA). Proteins were transferred to Amersham Protran 0.45-µm nitrocellulose membranes (Amersham, UK) before blocking for 1 h at room temperature in TBS-Tween 20 (0.05%) (TBS-T) and 5% non-fat milk. Primary antibodies were incubated overnight at 4 °C in TBS-T with 5% non-fat milk, and HRP-conjugated secondary antibodies were incubated for 1 h at room temperature. A list of the antibodies and dilutions can be seen in Table S10.

## 5. Conclusions

The present study has demonstrated that the use of in vitro models of sunitinib resistance, combined with an integrated genomic approach of miRNA and gene expression, can identify divergent mechanisms of sunitinib resistance that could be exploited for the benefit of ccRCC patients.

## Figures and Tables

**Table 1:** IC_50_ values of parental and sunitinib-resistant clones c1 and c2 generated from 786-O, A498, and Caki-1 cell lines as measured by MTT assay.

			IC_50_ Value	
Cell Line	Biological Replicate	Parental	Clone c1 (*p*-Value)	Clone c2 (*p*-Value)
786-O	A	5.7	17.32	13.54
	B	11.39	23.3	20.07
	C	6.7	20.4	12.8
	Average	7.93	20.34 **	15.47 *
A498	A	10.48	11.92	14.36
	B	7.682	12.42	16.04
	C	7.18	12.01	10.54
	Average	8.44	12.12 *	13.64 *
Caki-1	A	9.5	16.8	18
	B	9	14.6	17.3
	C	12.2	16.2	14.7
	Average	10.2	15.87 **	16.67 *

Values of biological triplicates are indicated by letters A–C. The dose response curves used to generate the IC_50_ values can be found in Figures S1–S3. *p*-values were calculated by an independent t-test and significance denoted by * *p* < 0.05, ** *p* < 0.01.

**Table 2:** List of differentially expressed genes (in both clones) targeted by differentially expressed miRNAs (in both clones) in sunitinib-resistant ccRCC cell lines.

Cell	miRNA/Gene	miRNA	Target Gene(s)
786-0	↑/↓	*hsa-miR-663a*	*CDKN1A*
	↑/↓	*hsa-miR-572*	*CDKN1A*
	↑/↓	*hsa-miR-638*	*PTEN*
	↑/↓	*hsa-miR-612*	*SP1*
	↑/↓	*hsa-miR-212-3p*	*RFXAP*
	↓/↑	*hsa-miR-106a-5p*	*SIRPA, MMP2, HIF1A*
	↓/↑	*hsa-miR-30d-5p*	*KPNB1, BNIP3L, BECN1*
	↓/↑	*hsa-miR-140-3p*	*ATP6AP2, FN1*
	↓/↑	*hsa-miR-26b-5p*	*PTGS2, ST8SIA4*
	↓/↑	*hsa-miR-17-5p*	*MMP2, SIRPA, GPR137B, EPAS1, VLDLR, HIF1A*
	↓/↑	*hsa-miR-200a-3p*	*WASF3,* *CD274*
	↓/↑	*hsa-miR-200b-3p*	*LOX, FN1, CD274, WASF3, MSN, FERMT2, FSCN1, RAB23*
	↓/↑	*hsa-miR-210-3p*	*HIF1A, BDNF, NCAM1, EHD2, TFRC*
	↓/↑	*hsa-miR-328-3p*	*PTPRJ, MMP16*
	↓/↑	*hsa-miR-34a-5p*	*BECN1, VAMP2, FUT8, INHBB, NOTCH2, MAGEA2, MAGEA3, L1CAM, AXL, PAM, SYT1, CD274*
	↓/↑	*hsa-miR-21-5p*	*BASP1, RECK, NFIB, SATB1, RHOB, PIAS3, DUSP10, LRP6*
	↓/↑	*hsa-miR-146a-5p*	*L1CAM, NOTCH2, PTGS2, HOXD10, RAC1*
	↓/↑	*hsa-miR-20a-5p*	*SIRPA, EPAS1, KIF26B, CRIM1, HIF1A, TSG101*
	↓/↑	*hsa-miR-17-3p*	*ITGA5, ITGB3*
	↓/↑	*hsa-miR-99a-5p*	*SMARCA5*
	↓/↑	*hsa-miR-18a-5p*	*PIAS3, TNFAIP3, HIF1A, TBPL1*
	↓/↑	*hsa-miR-25-5p*	*PRKCZ*
A498	↑/↓	*hsa-miR-34c-5p*	*ITPR1, HNF4A*
	↓/↑	*hsa-miR-145-5p*	*ITGB8, CTGFL, VPS51, EGFR*
Caki-1	↑/↓	*hsa-miR-148b-3p*	*SLC2A1*
	↑/↓	*hsa-miR-192-5p*	*ITGB3, ITGAV, CAV1, WNK1*
	↑/↓	*hsa-miR-29b-3p*	*DNMT3B*
	↓/↑	*hsa-miR-138-5p*	*CCND1*
	↓/↑	*hsa-miR-193b-3p*	*CCND1, AKR1C2*
	↓/↑	*hsa-miR-92a-5p*	*KLF2*
	↓/↑	*hsa-miR-296-3p*	*ICAM1*
	↓/↑	*hsa-miR-106b-5p*	*BMP2, RND3, CCND1*
	↓/↑	*hsa-miR-106b-3p*	*BMP2*
	↓/↑	*hsa-miR-130b-3p*	*IRF1*
	↓/↑	*hsa-miR-708-5p*	*CCND1*
	↓/↑	*hsa-miR-18a-5p*	*TNFAIP3, CTGF*
	↓/↑	*hsa-miR-17-5p*	*CCND1, BMP2, TCEAL1, RND3*
	↓/↑	*hsa-miR-1180-3p*	*TCEAL1*
	↓/↑	*hsa-miR-550a-5p*	*CPEB4*

**Table 3:** Densitometry readings of protein expression showing adjusted densities (%) relative to parental-control cell line.

Cell Line	786-O P	786-O c1	786-O c2	Caki-1 P	Caki-1 c1	Caki-1 c2	A498 P	A498 c1	A498 c2
PD-L1	100	161.3	131.3	100	16.9	35.8	100	1599.3	966.4
HIF1α	ND	ND	ND	ND	ND	ND	100	18.3	14.8
Cyclin D1	100	119.9	77.5	100	56.5	34.9	100	122.0	338.8
HIF2α	100	45.8	24.2	ND	ND	ND	100	128.5	60.8

Quantified using ImageJ software (v.1.8.0) (NIH, Bethesda, MD, USA) and expression adjusted according to respective loading controls using modified ImageJ protocol (http://www.lukemiller.org/ImageJ_gel_analysis.pdf, accessed on 10 August 2021). ND; not detected (i.e., raw value less than 50).

**Table 4:** Densitometry readings of protein expression showing adjusted densities (%) relative to Scramble-control.

Cell Line	P-scr	P-si	c1-scr	c1-P	c2-scr	c2-P
A498	100	0	100	15.8	100	4.5
786-O	100	35.7	100	28.5	100	20.2

Quantified using ImageJ software (v.1.8.0) (NIH, Bethesda, MD, USA) and expression adjusted according to respective loading controls using modified ImageJ protocol (http://www.lukemiller.org/ImageJ_gel_analysis.pdf, accessed on 10 August 2021).

**Table 5:** IC_50_ values of parental and sunitinib-resistant clones, c1 and c2, of cell lines 786-O, A498, and Caki-1 cell lines after treatment with either anti-*CD274* siRNA or a scramble RNA control (SCR).

Cell Line	Replicate	P-SCR	P-siRNA	c1-SCR	c1-siRNA	c2-SCR	c2- siRNA
786-O	A	5.9	4.2	11.0	6.5	10.6	6.6
	B	3.8	4.9	11.7	9.6	8.2	6.5
	C	4.7	6.8	12.3	9.5	9.4	8.0
	Average	4.8	5.3	11.7	8.5 *	9.4	7.0 *
A498	A	5.5	6.31	6.7	7.7	9.6	13.3
	B	9.8	11.8	10.6	12.8	10.2	11.4
	C	14.1	14.8	13	15.4	15.3	20.6
	Average	9.8	11	10.1	12	11.7	15.1
Caki-1	A	9.3	10.8	9	11.4	10	11.5
	B	6.4	6.7	9.5	16.1	11	11.9
	C	9.7	9.2	11	11.4	12	12.4
	Average	8.4	8.9	9.8	13	11	11.9

Values of biological triplicates are indicated by letters A–C. The dose curves used to generate the IC_50_ values can be found in Figures S6–S8. *p*-values were calculated by comparing siRNA-treated cells with SCR-treated cells by independent t-test and significance denoted by * *p* < 0.05.

## Data Availability

Data available in a publicly accessible repository. The data presented in this study are openly available in GEO at https://www.ncbi.nlm.nih.gov/geo/, accessed on 26 August 2021, GSE183140.

## Supplementary Materials

The following are available online at https://www.mdpi.com/article/10.3390/cancers13174401/s1, Figure S1: Cell proliferation MTT assays of 786-O resistant clones (c1 and c2) and the parental cell line used to calculate the IC_50_ value in Table 1. Figure S2: Cell proliferation MTT assays of A498 resistant clones (c1 and c2) and the parental cell line used to calculate the IC_50_ value in Table 1. Figure S3: Cell proliferation MTT assays of Caki-1 resistant clones (c1 and c2) and the parental cell line used to calculate the IC_50_ value in Table 1. Figure S4: Unsupervised cluster analysis heatmaps of miRNA expression (**A–C**) in 786-O (**A**), A498 (**B**) and Caki-1 (**C**) resistant clones and parental control cell lines. Figure S5: Relative *CD274* mRNA expression measured by qRT-PCR in sunitinib resistant clones, c1 and c2, and parental cells in 786-O (**A**), A498 (**B**) and Caki-1 (**C**) cell lines at 48 and 72 h post-transfection with either siRNA or a scramble control. Figure S6: Cell proliferation MTT assays of 786-O resistant clones (c1 and c2) and the parental cell line transfected with CD274-siRNA or scramble control siRNA. These data were used to calculate the IC_50_ value in Table 3. Figure S7: Cell proliferation MTT assays of A498 resistant clones (c1 and c2) and the parental cell line transfected with CD274-siRNA or scramble control siRNA. Figure S8: Cell proliferation MTT assays of Caki-1 resistant clones (c1 and c2) and the parental cell line transfected with CD274-siRNA or scramble control siRNA. Table S1: List of miRNAs commonly differentially expressed between 786-O parental (P) and resistant clones c1 and c2. Table S2: List of miRNAs commonly differentially expressed between A498 parental (P) and resistant clones c1 and c2. Table S3: List of miRNAs commonly differentially expressed between Caki-1 parental (P) and resistant clones c1 and c2. Table S4: List of top 100 differentially expressed genes between 786-O parental (P) and resistant clones c1 and c2. Arranged according to FDR *F-value*. Table S5: List of top 100 differentially expressed genes between A498 parental (P) and resistant clones c1 and c2. Arranged according to FDR *F-value*. Table S6: List of top 100 differentially expressed genes between Caki-1 parental (P) and resistant clones c1 and c2. Arranged according to FDR *F-value*. Table S7: List of significantly enriched KEGG pathways for 786-O cell line. Table S8: List of significantly enriched KEGG pathways for A498 cell line. Table S9: List of significantly enriched KEGG pathways for Caki-1 cell line. Table S10: List of primary and secondary antibodies used in the Western Blots.
