# Supplementary material for: Integrated mRNA and miRNA Transcriptomic Analyses Reveals Divergent Mechanisms of Sunitinib Resistance in Clear Cell Renal Cell Carcinoma (ccRCC)
Source: Cancers (Basel). 2021 Aug 31;13(17):4401. doi: 10.3390/cancers13174401 (PMC8430814; doi:10.3390/cancers13174401)
Supplement: Supplementary file 1 [file cancers-13-04401-s001.zip › cancers-1345736-supplementary.pdf]

cancers

MDPI

Supplementary Materials: Integrated mRNA and miRNA Transcriptomic Analyses Reveals Divergent Mechanisms of Sunitinib Resistance in Clear cell Renal Cell Carcinoma (ccRCC)

María Armesto, Maitane Marquez, María Arestin, Peio Errarte, Ane Rubio, Lorea Manterola, Jose I. López and Charles H. Lawrie

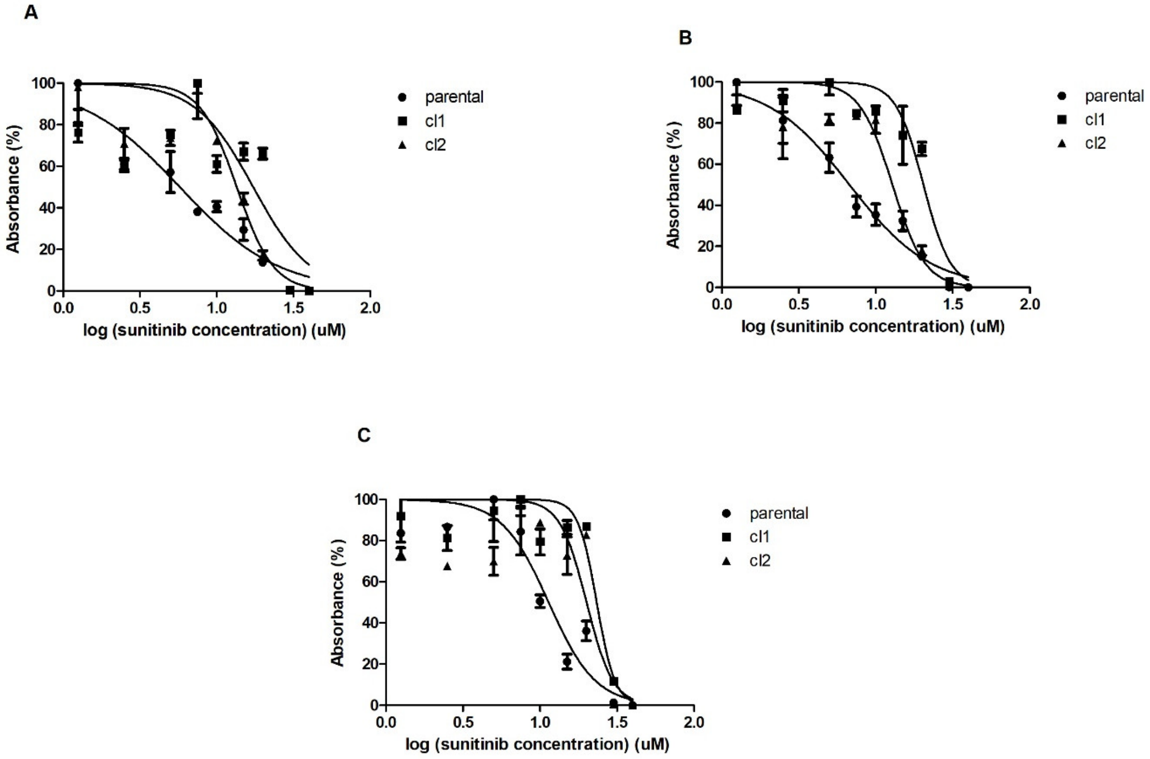

Figure S1. Cell proliferation MTT assays of 786–O resistant clones (c1 and c2) and the parental cell line used to calculate the IC_{50} value in Table 1. Biological replicate studies were carried out and are depicted in the respective graphs (**A**–**C**). Error bars were calculated from the readings obtained in technical triplicates.

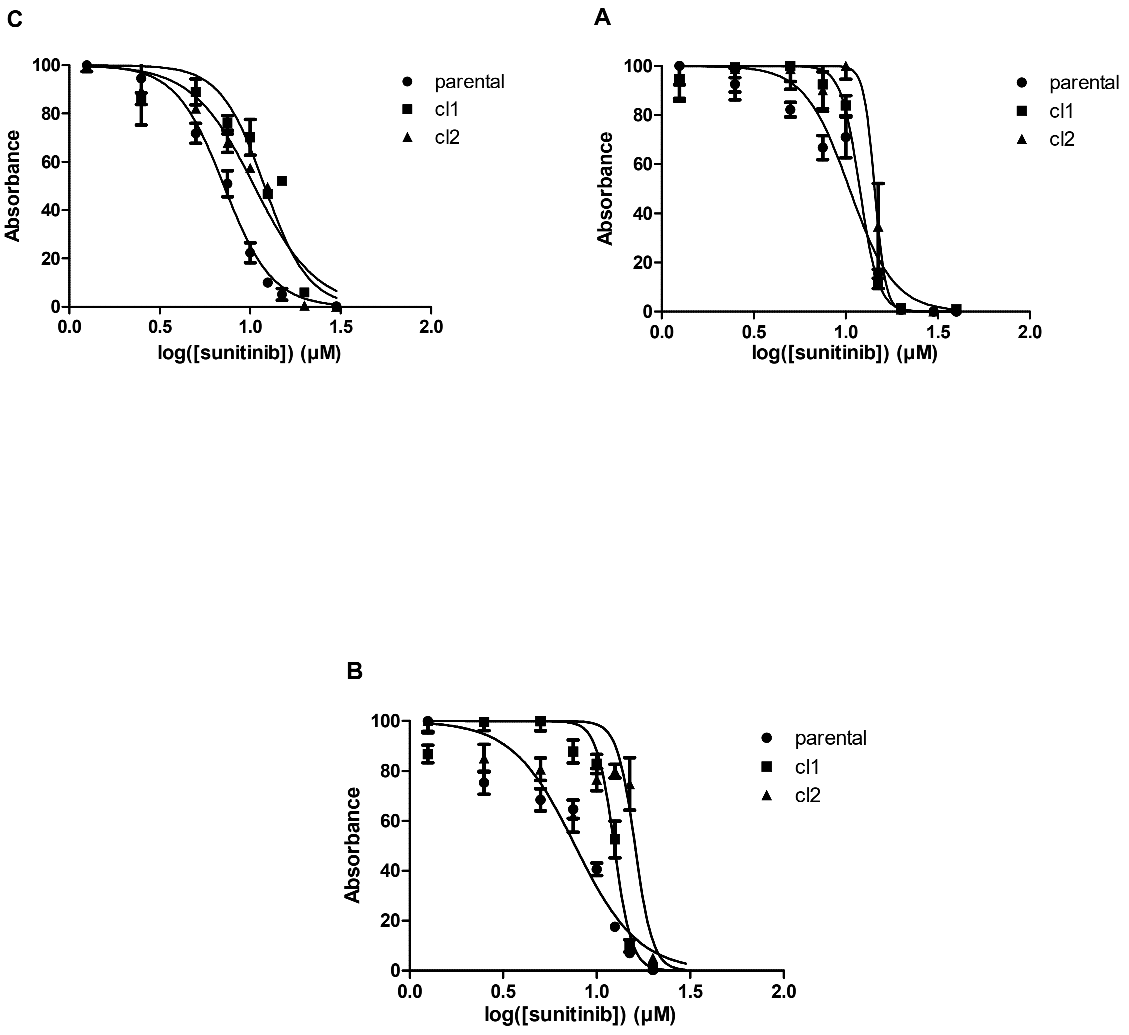

Figure S2. Cell proliferation MTT assays of A498 resistant clones (c1 and c2) and the parental cell line used to calculate the IC_{50} value in Table 1. Biological replicate studies were carried out and are depicted in the respective graphs (**A**–**C**). Error bars were calculated from the readings obtained in technical triplicates.

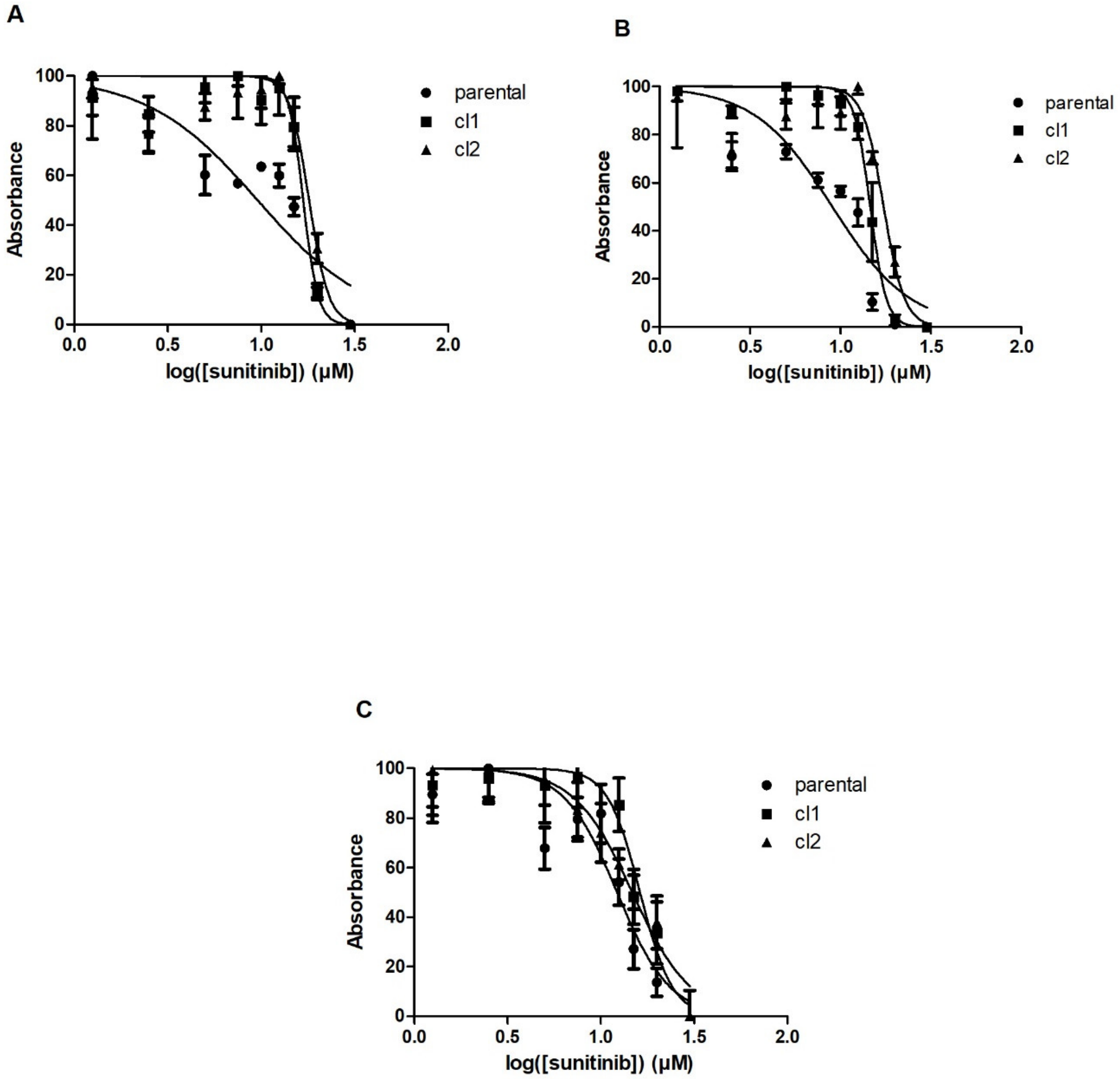

Figure S3. Cell proliferation MTT assays of Caki−1 resistant clones (c1 and c2) and the parental cell line used to calculate the IC_{50} value in Table 1. Biological replicate studies were carried out and are depicted in the respective graphs (**A**–**C**). Error bars were calculated from the readings obtained in technical triplicates.

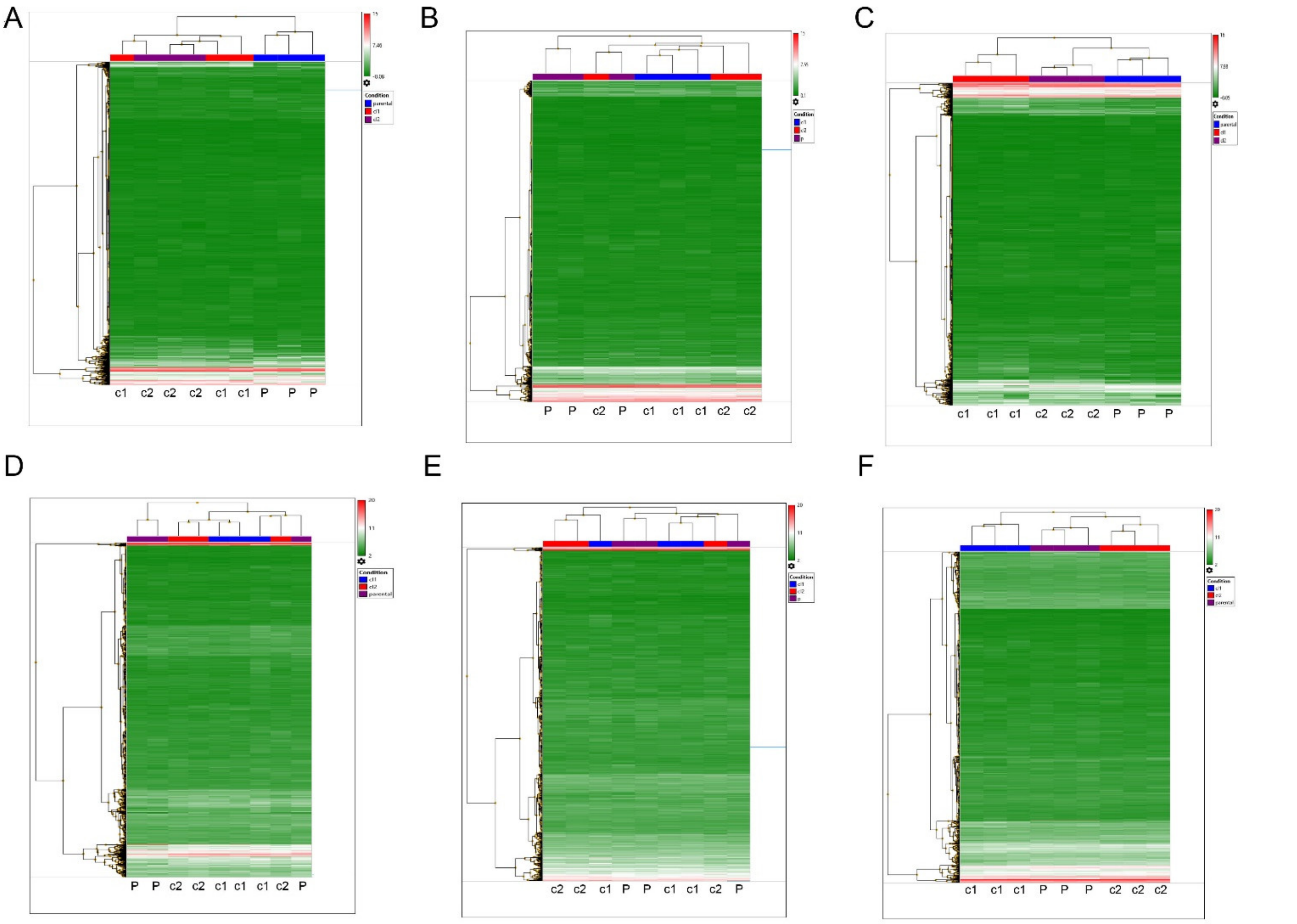

Figure S4. Unsupervised cluster analysis heatmaps of miRNA expression (**A–C**) in 786−O (**A**), A498 (**B**) and Caki−1 (**C**) resistant clones and parental control cell lines. Unsupervised cluster analysis heatmaps of gene expression (**D–F**) in 786−O (**D**), A498 (**E**) and Caki−1 (**F**) resistant clones and parental control cell lines.

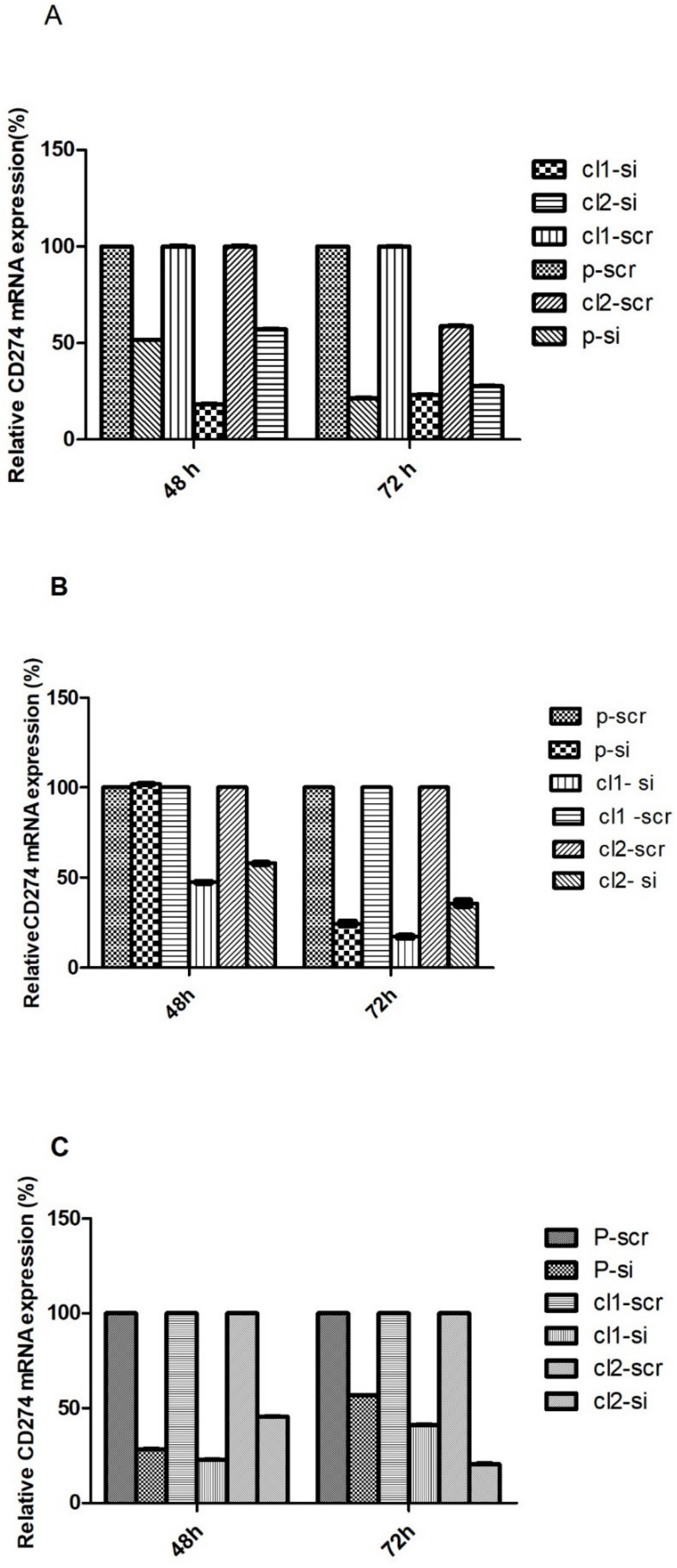

Figure S5. Relative *CD274* mRNA expression measured by qRT−PCR in sunitinib resistant clones, cl1 and cl2, and parental cells in 786 −O (**A**), A498 (**B**) and Caki−1 (**C**) cell lines at 48 and 72 h post−transfection with either siRNA or a scramble control.

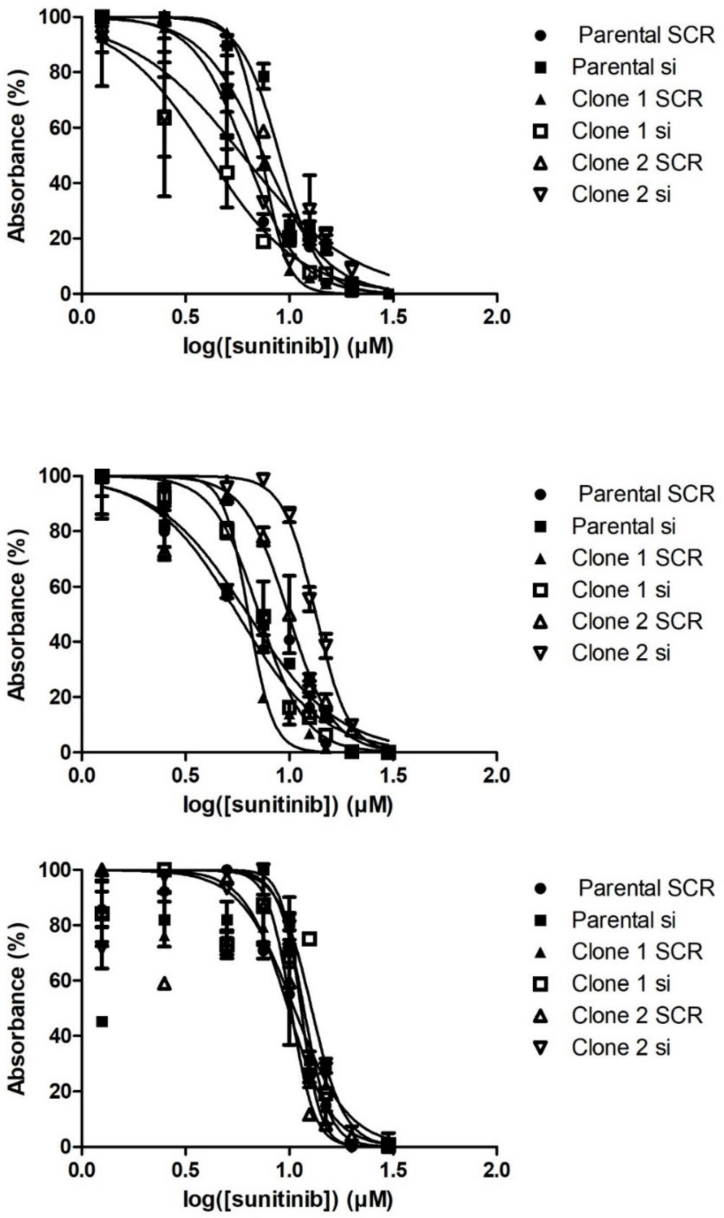

Figure S6. Cell proliferation MTT assays of 786–O resistant clones (c1 and c2) and the parental cell line transfected with CD274–siRNA or scramble control siRNA. These data were used to calculate the IC_{50} value in Table 3. Biological replicate studies were carried out and are depicted in the respective graphs (**A–C**). Error bars were calculated from the readings obtained in technical triplicates.

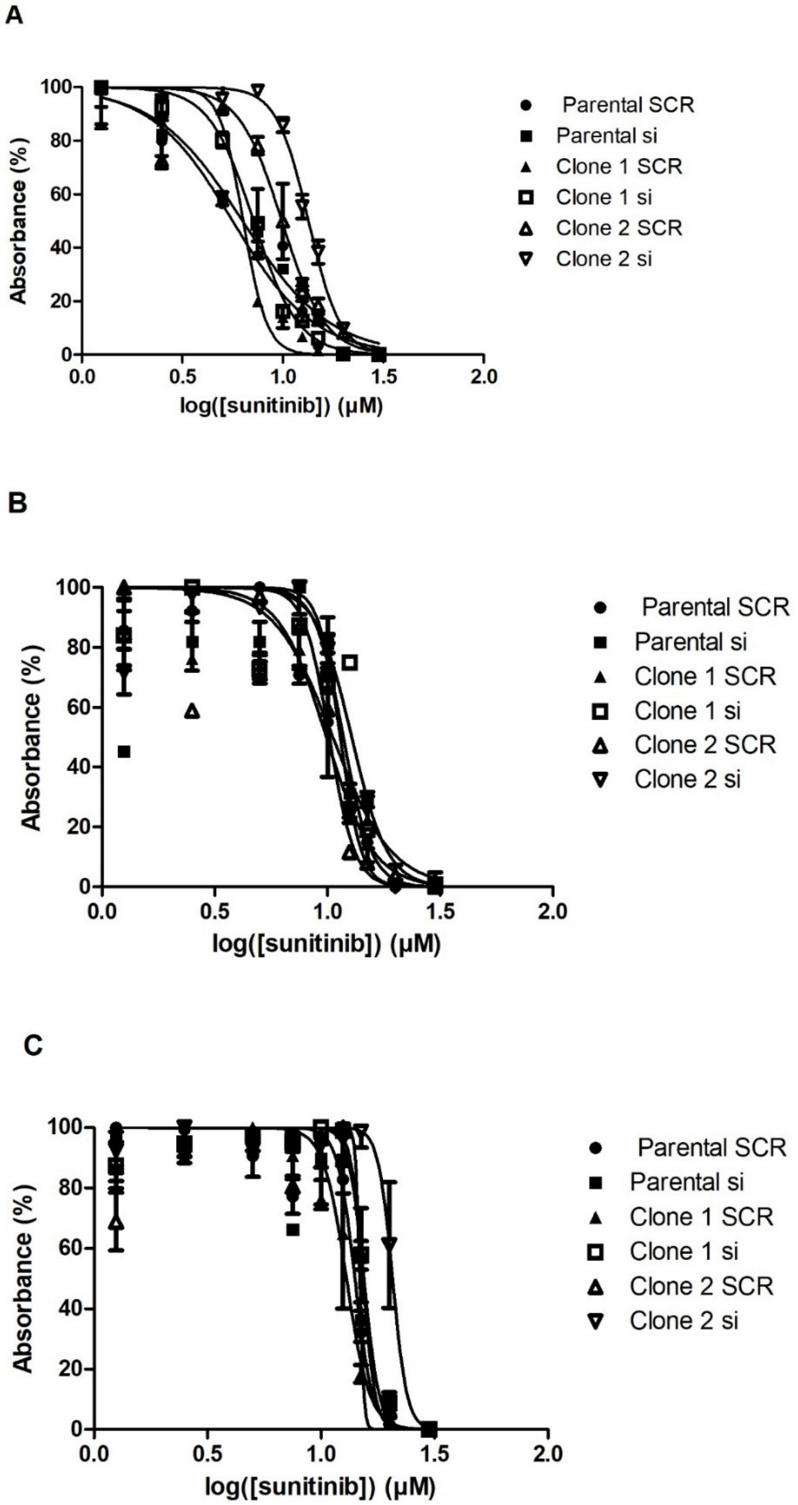

Figure S7. Cell proliferation MTT assays of A498 resistant clones (c1 and c2) and the parental cell line transfected with CD274–siRNA or scramble control siRNA. These data were used to calculate the IC_{50} value in Table 3. Biological replicate studies were carried out and are depicted in the respective graphs (**A–C**). Error bars were calculated from the readings obtained in technical triplicates.

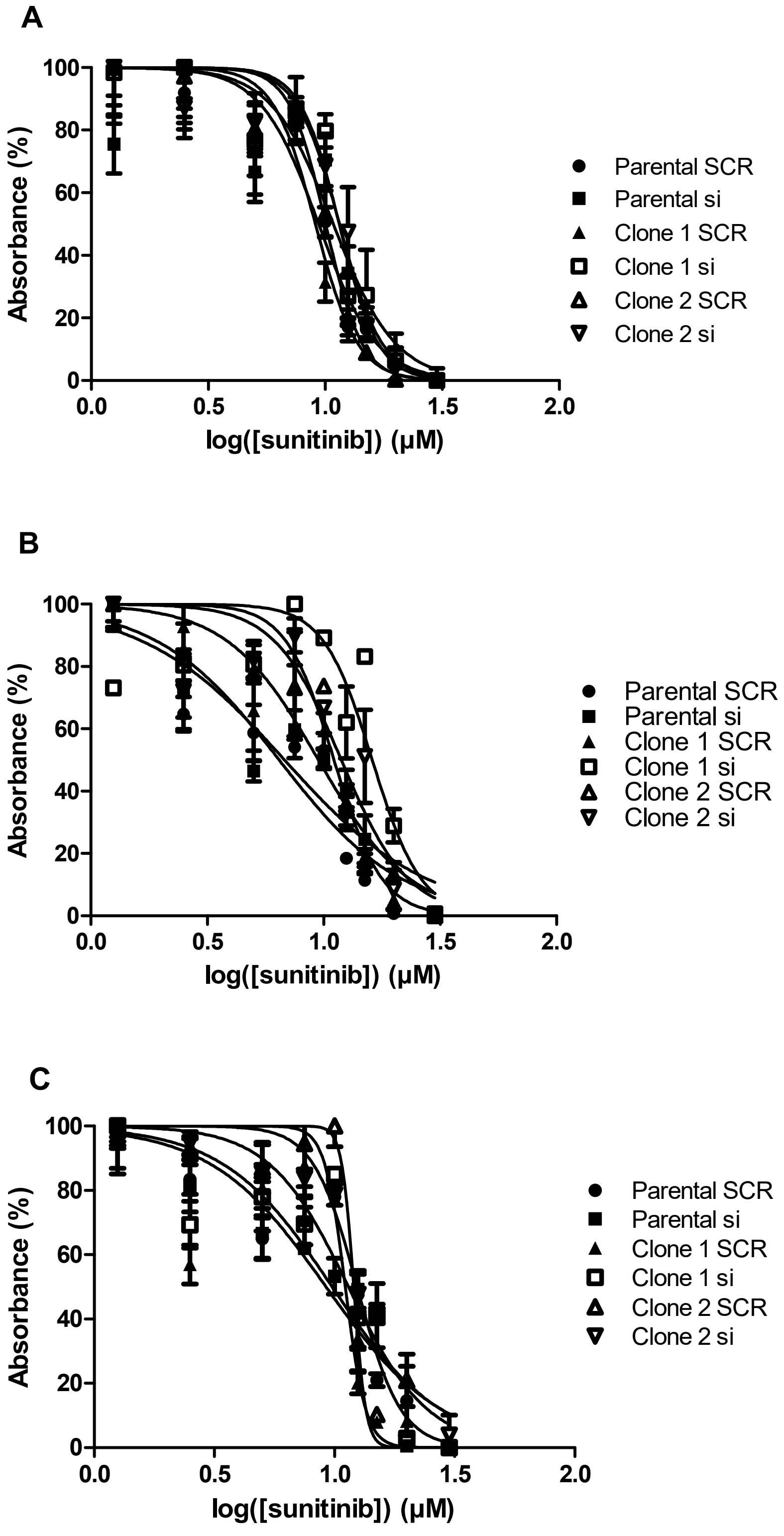

Figure S8. Cell proliferation MTT assays of Caki−1 resistant clones (c1 and c2) and the parental cell line transfected with CD274−siRNA or scramble control siRNA. These data were used to calculate the IC_{50} value in Table 3. Biological replicate studies were carried out and are depicted in the respective graphs (**A**–**C**). Error bars were calculated from the readings obtained in technical triplicates.

Table S1. List of miRNAs commonly differentially expressed between 786-O parental (P) and resistant clones c1 and c2.

miRNA	Clone1(c1)	Clone2(c2)	Parental (P)	c1/P (Fold)	c2/P(Fold)
hsa-miR-6068	181.02	222.86	1.62	111.14	137.01
hsa-miR-4467	1200.98	1217.75	18.77	64.25	65.22
hsa-miR-5001-5p	643.59	680.29	13.64	47.27	50.12
hsa-miR-642a-3p	118.60	105.42	2.93	40.48	36.01
hsa-miR-6825-5p	51.27	49.18	1.75	29.18	28.00
hsa-miR-1271-5p	43.71	10.85	2.03	21.63	5.36
hsa-miR-572	33.82	57.28	1.64	20.57	35.00
hsa-miR-7108-5p	1795.29	1722.16	89.88	19.90	19.15
hsa-miR-3937	38.59	42.22	2.20	17.48	19.10
hsa-miR-3621	61.39	66.26	4.50	13.61	14.67
hsa-miR-6754-5p	28.64	13.27	2.10	13.58	6.29
hsa-miR-6075	33.59	55.72	2.53	13.29	22.05
hsa-miR-4721	39.12	26.35	2.97	13.19	8.85
hsa-miR-642b-3p	58.08	72.50	4.76	12.15	15.25
hsa-miR-4651	232.32	184.82	20.11	11.55	9.22
hsa-miR-2861	2401.97	2336.28	222.86	10.80	10.53
hsa-miR-3154	21.86	23.59	2.03	10.75	11.65
hsa-miR-6860	20.82	8.57	2.06	10.09	4.15
hsa-miR-371b-5p	17.27	28.25	1.72	10.05	16.48
hsa-mir-6860	36.76	25.46	3.66	10.04	6.95
hsa-miR-8072	2778.33	2538.92	284.05	9.76	8.94
hsa-miR-638	**3848.29**	**3640.70**	**396.18**	**9.74**	**9.16**
hsa-miR-3911	40.50	24.59	4.20	9.69	5.87
hsa-miR-4649-5p	69.07	70.03	7.46	9.21	9.34
hsa-miR-4800-3p	16.68	22.01	1.89	8.85	11.61
hsa-miR-6126	374.81	407.31	42.52	8.82	9.58
hsa-miR-4430	65.34	73.01	7.46	8.74	9.79
hsa-miR-3180	23.26	18.00	2.69	8.62	6.69
hsa-miR-1915-3p	5042.77	4672.57	592.22	8.51	7.91
hsa-miR-935	17.75	12.21	2.10	8.49	5.83
hsa-miR-612	17.27	8.94	2.06	8.43	4.34
hsa-miR-6870-5p	32.22	29.86	3.84	8.40	7.82
hsa-miR-6802-5p	18.64	22.78	2.25	8.26	10.12
hsa-miR-6831-5p	62.25	41.93	8.00	7.79	5.25
hsa-miR-6088	5955.47	4544.80	770.69	7.72	5.88
hsa-miR-6132	27.67	24.76	3.61	7.68	6.91
hsa-mir-5703	54.95	82.71	7.31	7.52	11.31
hsa-miR-4486	12.73	7.67	1.73	7.38	4.44
hsa-miR-6125	5996.90	5914.33	843.36	7.14	7.01
hsa-mir-6090	20.39	16.34	2.87	7.13	5.68
hsa-miR-4436b-5p	15.03	11.00	2.17	6.91	5.05
hsa-miR-1908-5p	1858.60	1640.59	276.28	6.73	5.94
hsa-miR-4532	560.28	390.72	83.87	6.66	4.66
hsa-miR-4497	7082.29	5042.77	1082.39	6.58	4.68
hsa-miR-6127	61.39	50.21	9.45	6.52	5.35
hsa-miR-6727-5p	3821.70	3420.52	608.87	6.30	5.64
hsa-miR-6848-5p	19.43	13.00	3.18	6.09	4.09
hsa-miR-1281	31.12	41.07	5.13	6.05	8.01
hsa-miR-6724-5p	1951.00	2019.80	333.14	5.86	6.09
hsa-miR-6789-5p	192.67	167.73	33.59	5.72	4.99
hsa-miR-4665-5p	18.77	25.11	3.32	5.66	7.58
hsa-miR-4669	75.06	41.64	13.45	5.57	3.09
hsa-miR-6729-5p	5556.65	5873.48	1002.93	5.53	5.87
hsa-miR-191-3p	9.58	4.86	1.75	5.45	2.77
hsa-miR-4488	2304.12	1964.57	430.54	5.35	4.54
hsa-miR-5189-5p	16.22	13.18	3.03	5.35	4.33
hsa-miR-4505	254.23	158.68	48.50	5.25	3.29
hsa-miR-4466	3691.52	2836.70	719.08	5.12	3.92
hsa-miR-1225-5p	64.45	70.03	12.82	5.02	5.47
hsa-miR-1343-5p	164.28	118.60	34.30	4.79	3.44
hsa-miR-6805-5p	433.53	421.68	90.51	4.79	4.67
hsa-miR-6787-5p	46.85	46.85	9.92	4.74	4.72
hsa-miR-7704	10809.4	8599.28	2272.40	4.74	3.77
hsa-miR-6728-5p	13.93	11.55	3.01	4.64	3.85
hsa-miR-3188	18.51	10.20	4.06	4.58	2.52

hsa-miR-4750-5p	13.18	18.13	2.87	4.58	6.31
hsa-mir-3656	21.71	24.25	4.86	4.49	5.00
hsa-miR-138-1-3p	49.87	73.52	11.24	4.46	6.54
hsa-miR-149-3p	1520.15	1031.12	340.14	4.46	3.04
hsa-miR-1207-5p	407.31	263.20	91.14	4.45	2.89
hsa-miR-4787-5p	13400.5	10660.9	3019.30	4.45	3.54
hsa-miR-4690-5p	13.18	14.12	2.97	4.43	4.75
hsa-miR-3665	11268.4	11268.4	2556.58	4.41	4.41
hsa-miR-6820-5p	16.22	10.27	3.71	4.38	2.78
hsa-miR-3180-3p	38.05	34.54	8.69	4.37	3.96
hsa-miR-6726-5p	7.46	5.54	1.71	4.37	3.24
hsa-miR-4463	224.41	263.20	52.35	4.29	5.01
hsa-miR-6812-5p	168.90	91.14	39.95	4.25	2.28
hsa-mir-6089-1	270.60	250.73	64.45	4.21	3.90
hsa-mir-6089-2	270.60	250.73	64.45	4.21	3.90
hsa-miR-6791-5p	382.68	433.53	90.51	4.21	4.79
hsa-miR-6780b-5p	29.24	42.81	7.06	4.15	6.08
hsa-miR-4674	97.01	91.77	23.43	4.14	3.91
hsa-miR-3197	19.84	32.00	4.92	4.02	6.52
hsa-miR-5010-5p	10.34	5.74	2.60	3.97	2.21
hsa-miR-1587	24.42	18.51	6.19	3.95	2.98
hsa-miR-6821-5p	218.27	198.09	55.72	3.94	3.57
hsa-miR-6765-5p	249.00	203.66	64.00	3.89	3.18
hsa-miR-6786-5p	1200.98	1136.20	308.69	3.88	3.67
hsa-miR-6891-5p	57.28	60.13	15.35	3.75	3.92
hsa-miR-3141	265.03	168.90	72.00	3.67	2.34
hsa-miR-1910-5p	48.17	28.64	13.27	3.64	2.16
hsa-miR-3620-5p	212.31	174.85	60.13	3.54	2.92
hsa-miR-4745-5p	256.00	317.37	74.03	3.47	4.30
hsa-miR-4507	44.63	42.22	12.91	3.45	3.28
hsa-miR-8063	8.63	9.71	2.53	3.42	3.83
hsa-miR-6798-5p	64.45	76.64	19.16	3.37	4.01
hsa-miR-8069	3875.05	4039.61	1152.06	3.36	3.49
hsa-mir-4281	124.50	89.26	37.53	3.32	2.37
hsa-miR-4433b-3p	113.77	112.21	34.30	3.31	3.27
hsa-miR-6089	5556.65	5077.84	1675.06	3.31	3.03
hsa-miR-1237-5p	1323.37	1200.98	401.71	3.29	2.98
hsa-miR-6782-5p	33.36	24.59	10.20	3.29	2.41
hsa-miR-6087	10809.4	7750.10	3304.00	3.28	2.35
hsa-miR-6763-5p	33.13	29.04	10.20	3.25	2.86
hsa-miR-135a-3p	7.94	4.92	2.45	3.24	2.01
hsa-miR-1227-5p	97.68	124.50	30.91	3.16	4.03
hsa-miR-4707-5p	182.28	225.97	58.08	3.12	3.87
hsa-miR-3178	699.41	910.17	229.13	3.07	3.99
hsa-miR-4459	274.37	196.72	89.88	3.06	2.19
hsa-miR-663a	617.37	670.92	202.25	3.04	3.31
hsa-miR-937-5p	58.89	57.68	19.29	3.03	2.98
hsa-miR-1909-5p	8.51	5.78	2.85	2.99	2.04
hsa-miR-4689	64.89	65.34	22.01	2.95	2.98
hsa-miR-4734	121.94	159.79	41.64	2.93	3.84
hsa-miR-6090	3541.14	4299.64	1217.75	2.91	3.53
hsa-miR-920	4.89	3.41	1.69	2.90	2.02
hsa-miR-6774-5p	8.22	6.06	2.85	2.89	2.13
hsa-miR-4741	32.90	43.41	11.47	2.87	3.79
hsa-miR-6850-5p	145.01	182.28	51.27	2.82	3.55
hsa-miR-3960	14766.9	13969.5	5256.91	2.81	2.65
hsa-miR-6749-5p	69.07	64.45	24.76	2.78	2.61
hsa-miR-6716-5p	37.01	29.45	13.55	2.74	2.18
hsa-miR-4758-5p	43.41	64.45	16.00	2.71	4.02
hsa-miR-4433-3p	33.59	40.79	12.47	2.69	3.27
hsa-miR-675-5p	5.74	10.34	2.16	2.67	4.81
hsa-miR-212-3p	5.24	5.28	1.97	2.65	2.67
hsa-miR-5196-5p	22.63	26.35	8.51	2.65	3.08
hsa-miR-3940-5p	340.14	421.68	130.69	2.60	3.23
hsa-miR-4695-5p	37.27	47.84	15.24	2.46	3.16
hsa-miR-3162-5p	31.12	36.50	12.82	2.43	2.85
hsa-miR-4270	153.28	183.55	63.12	2.42	2.90
hsa-miR-6816-5p	349.71	374.81	145.01	2.42	2.60
hsa-miR-6808-5p	32.90	29.04	13.64	2.41	2.13

hsa-mir-4734	4.69	4.69	1.95	2.40	2.40
hsa-miR-6879-5p	58.08	63.12	24.08	2.40	2.61
hsa-miR-6752-5p	159.79	199.47	67.18	2.38	2.97
hsa-miR-6734-5p	8.28	10.41	3.51	2.35	2.96
hsa-miR-874-3p	3.89	4.41	1.72	2.26	2.56
hsa-mir-6790	10.20	9.58	4.59	2.23	2.09
hsa-miR-1229-5p	9.45	13.64	4.32	2.19	3.17
hsa-miR-6768-5p	8.69	12.13	4.20	2.08	2.89
hsa-miR-4783-3p	3.68	6.50	1.80	2.04	3.61
hsa-miR-940	9.78	10.34	4.82	2.03	2.15
hsa-miR-1469	617.37	760.08	306.55	2.02	2.49
hsa-miR-106a-5p	**996.00**	**792.35**	**2352.53**	**−2.36**	**−2.98**
Has-miR-17-5p	**1082.39**	**1112.82**	**2896.31**	**−2.67**	**−2.61**
hsa-miR-296-3p	34.78	32.22	100.43	−2.89	−3.11
hsa-miR-20a-5p	498.00	526.39	1448.15	−2.90	−2.75
hsa-miR-18a-3p	19.16	13.36	56.10	−2.94	−4.20
hsa-miR-4417	7.26	6.36	22.63	−3.12	−3.56
hsa-miR-3607-5p	1.67	2.04	5.82	−3.48	−2.85
hsa-miR-30d-5p	137.19	225.97	491.14	−3.60	−2.19
hsa-miR-18a-5p	**137.19**	**89.88**	**498.00**	**−3.64**	**−5.53**
hsa-miR-99a-5p	148.06	36.25	576.03	−3.91	−15.88
hsa-miR-4657	1.73	2.20	6.77	−3.92	−3.08
hsa-miR-93-5p	1530.73	1686.71	5996.90	−3.92	−3.55
hsa-miR-26b-5p	1.79	1.84	7.62	−4.25	−4.13
hsa-miR-140-3p	261.38	250.73	1192.69	−4.55	−4.74
hsa-miR-6511a-5p	2.95	6.50	13.74	−4.67	−2.13
hsa-miR-664b-5p	2.68	2.38	13.45	−5.05	−5.64
hsa-miR-4284	51.98	68.59	274.37	−5.27	−3.99
hsa-miR-328-3p	20.11	35.02	107.63	−5.32	−3.06
hsa-miR-25-5p	28.25	37.79	158.68	−5.63	−4.20
hsa-mir-21	**7.94**	**9.00**	**53.82**	**−6.77**	**−5.99**
hsa-miR-181a-3p	5.50	13.00	41.64	−7.59	−3.20
hsa-mir-3651	5.54	4.50	43.11	−7.76	−9.55
hsa-miR-1254	3.36	3.25	26.17	−7.80	−8.05
hsa-miR-4668-5p	2.53	1.65	22.32	−8.79	−13.48
hsa-miR-21-5p	**57.68**	**113.77**	**548.75**	**−9.53**	**−4.82**
hsa-miR-331-5p	2.81	3.61	27.47	−9.81	−7.67
hsa-miR-7641	45.25	79.34	461.44	−10.24	−5.84
hsa-miR-3651	93.05	109.90	1120.56	−12.03	−10.15
hsa-miR-7975	5.82	15.56	75.58	−12.99	−4.84
hsa-miR-17-3p	5.10	6.59	75.06	−14.73	−11.45
hsa-miR-21-3p	12.73	14.72	227.54	−17.86	−15.43
hsa-miR-34a-5p	**21.86**	**42.52**	**694.58**	**−31.73**	**−16.39**
hsa-miR-196b-3p	11.00	36.25	448.82	−40.93	−12.40

Values shown are median intensities. Those miRNAs that were validated by qRT-PCR are depicted in bold type.

Table S2. List of miRNAs commonly differentially expressed between A498 parental (P) and resistant clones c1 and c2.

miRNA	Clone1(c1)	Clone2(c2)	Parental	c1/P (Fold)	c2/P (Fold)
hsa-miR-6068	97.68	183.55	6.45	15.16	28.44
hsa-miR-371b-5p	18.13	14.12	2.35	7.74	6.02
hsa-miR-6126	487.75	354.59	72.00	6.78	4.91
hsa-miR-4467	415.87	760.08	70.52	5.88	10.76
hsa-miR-1202	19.56	10.63	3.34	5.84	3.17
hsa-miR-34c-5p	43.11	21.11	8.69	4.95	2.43
hsa-miR-4430	42.52	20.11	9.13	4.65	2.2
hsa-miR-6754-5p	12.13	9.71	2.71	4.46	3.57
hsa-miR-6075	32.67	29.65	8.34	3.9	3.56
hsa-miR-150-3p	43.71	51.98	11.16	3.9	4.64
hsa-miR-7108-5p	786.88	1144.10	206.50	3.81	5.54
hsa-miR-3162-5p	37.01	24.76	10.85	3.41	2.29
hsa-miR-5001-5p	436.55	588.13	133.44	3.29	4.41
hsa-miR-15-5p	**1152.06**	**1448.15**	**354.59**	**3.24**	**4.09**
hsa-miR-1915-3p	2179.83	3191.46	704.28	3.1	4.52
hsa-miR-4505	124.50	129.79	40.79	3.06	3.18
hsa-miR-6724-5p	861.08	1234.75	284.05	3.04	4.36
hsa-miR-4787-5p	4672.57	6038.61	1584.71	2.96	3.82
hsa-miR-4689	78.25	82.14	26.91	2.91	3.05
hsa-miR-2861	968.76	1217.75	330.84	2.91	3.68
hsa-miR-6743-5p	433.53	357.05	151.17	2.86	2.36
hsa-miR-3937	30.70	29.86	10.70	2.86	2.78
hsa-miR-8072	1097.50	1746.20	388.02	2.82	4.49
hsa-miR-4741	49.18	86.22	18.00	2.74	4.79
hsa-miR-6729-5p	2336.28	3468.27	855.13	2.73	4.06
hsa-miR-34c-3p	58.49	48.84	21.71	2.69	2.25
hsa-miR-638	**1509.65**	**2210.26**	**564.18**	**2.66**	**3.9**
hsa-miR-6132	21.41	23.10	8.11	2.64	2.86
hsa-miR-1225-5p	51.98	73.52	19.84	2.62	3.69
hsa-miR-6125	2418.67	3615.55	962.07	2.51	3.77
hsa-miR-4516	1584.71	1562.89	634.73	2.51	2.48
hsa-miR-3665	5556.65	6793.79	2336.28	2.39	2.91
hsa-miR-1908-5p	879.17	1458.23	369.65	2.37	3.93
hsa-miR-320e	168.90	141.04	344.89	−2.04	−2.45
hsa-miR-484	119.43	122.79	252.48	−2.11	−2.05
hsa-miR-550a-3p	99.73	82.71	219.79	−2.22	−2.65
hsa-miR-671-3p	57.28	55.33	128.00	−2.23	−2.32
hsa-miR-99a-5p	855.13	652.58	1910.85	−2.23	−2.92
hsa-miR-125b-2-3p	76.11	65.80	184.82	−2.42	−2.79
hsa-miR-148b-3p	15.14	10.06	36.76	−2.43	−3.65
hsa-miR-363-3p	1.68	1.47	4.14	−2.45	−2.81
hsa-mir-92b	121.94	151.17	304.44	−2.5	−2.01
hsa-miR-34a-3p	**77.71**	**78.25**	**195.36**	**−2.5**	**−2.49**
hsa-miR-2110	26.54	13.93	67.18	−2.53	−4.82
hsa-miR-1296-5pç	13.18	16.68	35.75	−2.72	−2.14
hsa-miR-1910-3p	4.00	5.39	12.55	−3.13	−2.33
hsa-miR-92b-3p	809.00	1269.46	2665.15	−3.28	−2.1
hsa-miR-146b-5p	**8.28**	**7.11**	**33.36**	**−4.04**	**−4.69**
hsa-miR-328-3p	4.56	15.03	48.17	−10.52	−3.19
hsa-miR-145-5p	1.83	2.19	20.53	−11.23	−9.42

Table S3. List of miRNAs commonly differentially expressed between Caki-1 parental (P) and resistant clones c1 and c2.

miRNA	Clone1(c1)	Clone2(c2)	Parental	c1/P (Fold)	c2/P(Fold)
miR-92a-1-5p	1428.22	328.56	2.79	512	118.07
miR-200b-5p	**382.68**	**118.60**	**2.95**	**129.76**	**40.04**
miR-200a-5p	**135.30**	**62.68**	**1.75**	**76.79**	**35.74**
miR-210-5p	**224.41**	**76.11**	**3.68**	**61.13**	**20.74**
miR-708-5p	156.50	19.70	2.71	57.66	7.27
miR-92b-3p	138.14	29.45	3.05	45.2	9.63
miR-193b-5p	256.00	33.59	6.36	40.05	5.25
miR-18a-3p	290.02	41.07	7.52	38.47	5.46
miR-210-3p	5914.33	1782.89	182.28	32.31	9.78
miR-3613-5p	55.33	4.99	1.87	29.72	2.68

miR-138-5p	46.85	10.13	1.93	24.32	5.26
miR-766-3p	53.45	18.77	2.22	24.11	8.47
miR-2110	64.00	19.84	2.75	23.32	7.22
mir-92b	74.54	97.01	3.29	22.75	29.46
miR-328-3p	59.30	17.63	2.71	21.93	6.5
miR-138-1-3p	54.95	16.22	2.77	19.9	5.87
miR-125a-3p	1192.69	232.32	61.82	19.34	3.75
miR-5189-3p	37.53	4.23	2.01	18.66	2.1
miR-1307-3p	81.57	24.25	4.38	18.63	5.52
miR-23a-5p	78.79	25.28	4.26	18.55	5.96
miR-3184-3p	133.44	40.50	7.21	18.51	5.61
miR-18a-5p	**85.63**	**27.67**	**4.66**	**18.42**	**5.93**
miR-365a-5p	580.04	105.42	31.78	18.35	3.33
miR-1180-3p	2876.30	803.41	156.50	18.35	5.13
miR-18b-5p	45.89	16.11	2.51	18.26	6.42
miR-365b-5p	93.70	41.36	5.28	17.8	7.85
miR-8075	344.89	125.37	19.84	17.39	6.32
miR-197-3p	112.99	40.50	7.26	15.52	5.58
miR-423-5p	31.34	8.94	2.20	14.27	4.06
miR-550a-3-5p	71.51	15.78	5.03	14.25	3.14
miR-1972	21.71	8.57	1.59	13.63	5.4
miR-193a-5p	39.12	19.43	2.89	13.5	6.72
miR-345-5p	83.87	28.25	6.19	13.49	4.54
miR-744-5p	34.54	10.56	2.58	13.33	4.06
miR-31-5p	30.70	8.46	2.35	13.13	3.6
miR-101-5p	352.14	95.01	26.91	13.03	3.51
miR-1301-3p	39.12	14.03	3.03	12.88	4.62
miR-941	63.12	18.90	4.99	12.64	3.77
miR-331-5p	19.70	9.25	1.58	12.45	5.85
miR-615-3p	52.35	19.97	4.35	12.06	4.58
miR-550a-5p	35.75	25.99	2.99	11.98	8.71
miR-455-3p	86.22	36.25	7.21	11.97	5.02
miR-330-3p	263.20	69.55	23.26	11.3	2.98
miR-877-5p	28.05	18.38	2.53	11.09	7.27
miR-30b-3p	61.39	32.45	5.58	11.04	5.83
miR-21-5p	**219.79**	**60.97**	**20.11**	**10.93**	**3.03**
miR-585-5p	49.52	22.32	4.53	10.91	4.93
miR-106a-5p	**30.48**	**33.36**	**2.83**	**10.8**	**11.79**
miR-1275	126.24	69.55	11.71	10.76	5.94
miR-4284	23.43	8.11	2.25	10.36	3.59
miR-4417	82.14	38.05	8.06	10.22	4.72
miR-17-5p	**69.55**	**27.47**	**6.92**	**10.06**	**3.95**
miR-652-3p	31.56	7.67	3.16	9.99	2.43
miR-505-5p	29.65	13.83	2.99	9.92	4.6
miR-125b-1-3p	35.75	48.50	3.68	9.69	13.17
miR-378a-3p	50.56	21.56	5.31	9.52	4.04
mir-6776	5404.70	1710.26	572.05	9.46	2.99
miR-574-3p	41.93	20.11	4.53	9.23	4.43
miR-4701-3p	14.83	5.03	1.64	9.03	3.07
miR-423-3p	57.28	32.22	6.45	8.88	4.98
miR-193b-3p	21.71	10.13	2.45	8.87	4.12
miR-339-5p	15.89	7.78	1.82	8.74	4.29
miR-874-3p	79.89	43.11	9.32	8.54	4.63
miR-130b-3p	24.25	13.36	2.89	8.44	4.63
miR-320a	16.11	7.73	1.96	8.22	3.93
miR-320b	74.54	33.36	9.06	8.2	3.67
miR-502-3p	7696.57	2256.70	942.27	8.17	2.4
miR-1343-3p	192.67	54.95	23.75	8.15	2.31
miR-296-3p	156.50	48.84	19.29	8.12	2.53
miR-106b-3p	19.70	13.36	2.48	7.94	5.4
miR-23b-5p	27.86	18.64	3.58	7.75	5.19
miR-2115-5p	36.25	19.29	4.69	7.7	4.09
miR-1296-5p	44.02	28.64	5.74	7.66	5.01
miR-31-3p	19.03	59.71	2.48	7.66	24.06
miR-491-5p	42.22	27.67	5.54	7.58	4.98
miR-27a-5p	26.17	71.01	3.48	7.56	20.44
miR-99b-3p	18.38	28.05	2.43	7.55	11.56
miR-25-5p	17.15	5.24	2.28	7.51	2.3
miR-339-3p	14.32	3.94	1.92	7.49	2.05

miR-769-3p	24.25	30.48	3.25	7.46	9.42
miR-320c	9877.98	2977.74	1332.57	7.45	2.24
miR-615-5p	26.91	10.93	3.61	7.44	3.02
miR-4298	306.55	135.30	42.22	7.29	3.22
mir-526a-2	48.50	19.70	6.82	7.12	2.89
miR-7847-3p	12.47	4.66	1.75	7.09	2.66
miR-93-5p	103.97	35.51	14.83	7.03	2.4
mir-4449	13.09	12.73	1.85	7.02	6.84
miR-3663-3p	261.38	86.22	37.79	6.89	2.28
mir-711	16.11	8.69	2.35	6.86	3.72
miR-6836-5p	102.54	35.26	15.24	6.74	2.33
miR-7846-3p	14.93	9.92	2.23	6.65	4.41
miR-181c-3p	11993.79	3640.70	1807.78	6.62	2.02
mir-4734	48.50	40.79	7.31	6.62	5.59
miR-3137	24.59	15.78	3.76	6.54	4.19
miR-4776-5p	17.75	9.71	2.71	6.54	3.57
miR-6084	25709.25	12077.21	3983.99	6.49	3.04
miR-1199-5p	28.84	21.11	4.50	6.43	4.69
miR-203a	21.56	9.45	3.36	6.42	2.82
miR-1238-5p	48.50	45.57	7.57	6.4	6.02
miR-4731-5p	14.03	5.24	2.19	6.39	2.39
miR-6856-5p	21.11	8.46	3.29	6.39	2.56
miR-6875-5p	121.10	38.59	18.90	6.37	2.04
mir-4674	16.56	7.84	2.62	6.3	2.97
miR-6076	38.32	122.79	6.11	6.27	20.02
miR-4458	13.93	25.46	2.23	6.25	11.4
miR-6735-5p	14.93	5.21	2.43	6.17	2.15
miR-224-5p	18.00	6.45	2.95	6.13	2.2
miR-4784	12.13	4.38	1.97	6.13	2.22
miR-192-5p	11190.60	4640.29	1820.35	6.12	2.55
miR-4253	18.51	7.16	3.05	6.07	2.35
miR-4725-3p	22.16	14.52	3.66	6.05	3.98
miR-3714	14.62	12.04	2.45	5.98	4.94
miR-8060	13587.57	5914.33	2288.20	5.93	2.59
miR-3194-5p	13682.08	6888.62	2320.15	5.88	2.96
mir-638	**8.22**	**3.53**	**1.39**	**5.88**	**2.53**
miR-4486	158.68	66.26	27.28	5.83	2.43
miR-4465	15.78	6.73	2.75	5.74	2.45
miR-183-3p	26.91	81.57	4.69	5.72	17.29
mir-3150a	108.38	41.07	19.56	5.56	2.1
miR-301a-3p	14.83	13.00	2.68	5.52	4.85
mir-3917	14.83	16.00	2.71	5.45	5.89
mir-8072	8.94	4.06	1.64	5.44	2.48
miR-8071	7.67	2.93	1.43	5.35	2.04
miR-6830-5p	55.33	24.59	10.41	5.32	2.37
miR-363-3p	10.06	7.94	1.91	5.29	4.17
miR-3622b-5p	11.96	65.80	2.28	5.24	28.76
miR-3187-3p	14.93	9.00	2.93	5.09	3.08
miR-6759-5p	22.47	15.03	4.44	5.05	3.38
miR-7977	60.13	31.56	12.21	4.91	2.59
miR-6873-5p	14.93	10.48	3.05	4.89	3.42
miR-1236-5p	216.77	110.66	44.94	4.82	2.46
miR-4788	8.63	3.73	1.79	4.82	2.09
miR-6738-5p	13.74	6.28	2.85	4.81	2.2
miR-126-3p	14.32	8.06	2.97	4.81	2.71
miR-5088-5p	18.51	8.00	3.86	4.78	2.07
mir-6790	14.22	48.50	2.99	4.77	16.25
miR-711	29.24	22.16	6.11	4.77	3.62
miR-4638-5p	21.11	11.96	4.44	4.77	2.7
miR-6080	498.00	337.79	104.69	4.75	3.23
miR-6746-3p	11.16	5.70	2.41	4.64	2.38
mir-611	21.71	11.16	4.69	4.6	2.37
miR-3156-5p	17.15	11.00	3.81	4.52	2.9
miR-1909-3p	105.42	48.84	23.43	4.49	2.09
miR-5189-5p	15.56	12.91	3.48	4.47	3.69
miR-4454	47.50	30.06	10.63	4.47	2.83
miR-4498	17.15	30.06	3.86	4.44	7.74
mir-4632	7.67	36.00	1.75	4.38	20.49
miR-6869-5p	84.45	39.95	19.43	4.37	2.05

mir-4787	15.45	16.91	3.53	4.36	4.78
miR-4688	70.03	40.22	16.00	4.35	2.51
miR-628-3p	10.27	5.35	2.41	4.26	2.23
miR-6879-5p	7.11	4.82	1.67	4.26	2.89
miR-7152-3p	174.85	82.71	41.36	4.24	2
miR-4695-3p	23.75	48.17	5.62	4.23	8.61
miR-6782-5p	59.30	51.63	14.03	4.22	3.7
miR-6845-5p	15.14	10.06	3.63	4.15	2.77
miR-584-5p	13969.57	7486.11	3373.43	4.14	2.22
miR-29b-3p	**6.41**	**4.69**	**1.55**	**4.13**	**3.04**
miR-4667-5p	45.57	34.30	11.08	4.11	3.1
miR-6777-5p	5595.30	6608.01	1379.57	4.05	4.8
miR-4655-5p	61.39	53.82	15.24	4.04	3.52
miR-8089	232.32	127.12	57.68	4.03	2.2
miR-3622a-5p	6.50	4.89	1.67	3.88	2.92
miR-6795-5p	5.35	2.87	1.39	3.85	2.06
miR-4708-5p	8.11	4.50	2.13	3.81	2.11
miR-3185	6.82	4.17	1.79	3.81	2.33
miR-22-5p	8.69	5.10	2.30	3.79	2.22
miR-4743-5p	6.77	5.82	1.79	3.78	3.24
mir-3960	59.30	32.90	15.89	3.75	2.07
miR-4539	9.58	11.00	2.57	3.74	4.28
miR-4665-3p	42.52	116.16	11.47	3.7	10.08
miR-4481	8.17	7.21	2.23	3.65	3.23
miR-4741	11.24	14.83	3.14	3.58	4.72
miR-4479	12.55	16.80	3.61	3.49	4.68
miR-6784-5p	809.00	929.30	232.32	3.47	3.98
miR-6510-5p	7.78	5.03	2.25	3.47	2.23
miR-6877-5p	7.78	5.50	2.36	3.29	2.33
miR-8085	18.51	20.25	5.66	3.25	3.57
miR-148a-3p	7.11	8.00	2.19	3.25	3.63
miR-6829-5p	7.94	46.53	2.48	3.22	18.85
miR-2392	7.62	9.25	2.38	3.21	3.89
mir-3123	12.21	10.78	3.86	3.17	2.78
miR-6772-5p	7.84	5.66	2.48	3.15	2.28
miR-6887-5p	4.03	3.32	1.29	3.1	2.56
miR-659-3p	5.13	11.08	1.68	3.06	6.58
miR-6848-5p	13.09	9.99	4.35	3	2.3
miR-660-5p	10.93	24.42	3.73	2.94	6.56
miR-4322	5.66	7.62	2.00	2.82	3.79
miR-4665-5p	79.34	73.01	28.25	2.81	2.59
miR-6729-5p	18.51	16.00	6.63	2.79	2.41
miR-6892-5p	4.56	5.50	1.66	2.75	3.3
miR-6125	12.38	12.64	4.53	2.73	2.79
miR-6740-5p	5.21	7.84	1.92	2.72	4.09
miR-665	6.92	7.67	2.55	2.71	3.01
miR-4513	4.35	4.29	1.62	2.68	2.64
miR-638	**19.70**	**188.71**	**7.41**	**2.66**	**25.46**
miR-4722-5p	3.58	2.97	1.37	2.61	2.17
miR-4257	8.46	47.50	3.25	2.61	14.7
miR-1304-3p	13.64	11.79	5.24	2.59	2.25
miR-34a-3p	8.17	12.73	3.18	2.56	4
miR-452-5p	8.22	11.88	3.34	2.47	3.56
miR-6737-5p	19.56	16.56	8.06	2.43	2.06
miR-5196-5p	5.98	13.00	2.51	2.39	5.19
miR-6800-3p	3.97	4.00	1.66	2.38	2.4
miR-6796-5p	4.35	3.86	1.85	2.34	2.1
miR-6086	4.35	4.69	1.87	2.34	2.51
miR-5572	109.90	357.05	47.84	2.3	7.46
miR-7106-5p	4.11	7.36	1.84	2.23	4
miR-4787-5p	4.66	4.66	2.10	2.22	2.22
miR-4685-5p	4.69	4.69	2.14	2.2	2.19
miR-4767	17.88	19.97	8.17	2.18	2.44
miR-3197	3.86	3.92	1.77	2.18	2.21
miR-1915-3p	8.69	25.11	4.17	2.08	6.02
miR-3682-3p	12.64	14.22	6.15	2.06	2.31
miR-4690-5p	33.82	33.82	68.12	−2.01	−2.01
miR-3652	50.21	39.95	101.13	−2.02	−2.53
miR-7150	184.82	182.28	385.34	−2.1	−2.12

miR-4442	166.57	141.04	357.05	−2.14	−2.54
mir-3656	2005.85	2105.58	4299.64	−2.15	−2.04
miR-8064	556.41	596.34	1251.98	−2.24	−2.1
miR-6861-5p	1.34	1.49	3.01	−2.25	−2.02
miR-4695-5p	410.15	448.82	935.76	−2.29	−2.09
miR-6833-5p	14.32	10.34	37.27	−2.61	−3.6
miR-8072	2957.17	3691.52	7750.10	−2.63	−2.1
miR-6721-5p	35.02	45.25	93.05	−2.66	−2.06
miR-4708-3p	170.07	182.28	455.09	−2.68	−2.5
miR-6760-5p	60.97	44.02	166.57	−2.73	−3.79
miR-1244	245.57	151.17	694.58	−2.84	−4.59
miR-148b-3p	86.82	124.50	257.78	−2.97	−2.06
miR-6781-5p	76.64	60.97	232.32	−3.03	−3.8
miR-140-5p	4.79	37.27	14.62	−3.04	2.55
miR-6728-5p	61.39	29.86	192.67	−3.14	−6.46
miR-8063	247.28	242.19	786.88	−3.19	−3.26
miR-483-5p	93.70	141.04	298.17	−3.19	−2.13
miR-3147	82.14	97.01	263.20	−3.2	−2.71
miR-3162-5p	4.92	3.84	16.22	−3.3	−4.25
miR-4433-3p	56.89	38.32	188.71	−3.3	−4.91
miR-2861	168.90	249.00	564.18	−3.33	−2.26
mir-5703	3902.01	4359.66	13124.73	−3.36	−3.01
mir-4785	4011.71	4544.80	13587.57	−3.38	−2.99
miR-6726-5p	207.94	263.20	704.28	−3.4	−2.68
miR-5006-5p	34.06	29.24	116.16	−3.41	−3.98
miR-211-3p	238.86	374.81	831.75	−3.48	−2.21
miR-6754-3p	1448.15	1287.18	5113.16	−3.54	−3.97
miR-5739	781.44	781.44	2876.30	−3.7	−3.7
mir-6729	10.06	14.32	37.53	−3.72	−2.63
mir-6869	1606.83	1782.89	5996.90	−3.74	−3.37
miR-7108-5p	6.36	9.58	23.92	−3.75	−2.49
miR-6799-5p	86.82	91.14	326.29	−3.77	−3.57
miR-6790-5p	280.14	224.41	1060.11	−3.77	−4.7
miR-346	53.45	73.52	206.50	−3.88	−2.82
miR-6734-5p	92.41	114.56	359.54	−3.89	−3.14
miR-6132	2538.92	3956.48	9946.68	−3.92	−2.52
miR-933	24.59	40.79	97.01	−3.94	−2.38
miR-1909-5p	186.11	215.27	739.29	−3.96	−3.43
miR-6075	288.01	128.89	1152.06	−3.98	−8.93
miR-885-3p	1964.57	3492.39	8023.41	−4.07	−2.29
miR-4640-5p	2.08	3.07	8.57	−4.13	−2.79
miR-1225-5p	242.19	380.04	1016.93	−4.19	−2.66
miR-6820-5p	28.25	38.59	120.26	−4.28	−3.13
miR-1202	91.77	91.14	401.71	−4.36	−4.4
miR-6794-5p	22.94	22.32	100.43	−4.37	−4.5
miR-6802-5p	22.47	37.79	102.54	−4.56	−2.72
miR-6746-5p	410.15	498.00	1871.53	−4.56	−3.76
miR-6741-5p	109.90	154.34	504.95	−4.6	−3.29
miR-4271	30.70	61.39	142.02	−4.61	−2.31
miR-6797-5p	36.50	34.78	168.90	−4.65	−4.86
miR-4428	57.28	46.53	268.73	−4.7	−5.75
miR-937-5p	14.52	13.83	68.12	−4.71	−4.94
miR-135a-3p	2592.27	4096.00	12330.98	−4.75	−3
miR-6894-5p	284.05	167.73	1360.57	−4.78	−8.14
miR-1587	128.00	171.25	625.99	−4.89	−3.67
miR-6774-5p	765.36	1112.82	3821.70	−4.98	−3.43
miR-943	14.03	26.91	71.01	−5.05	−2.63
miR-3188	12.30	30.27	62.68	−5.07	−2.06
miR-1238-3p	625.99	430.54	3236.01	−5.15	−7.53
miR-4800-3p	143.01	272.48	744.43	−5.21	−2.74
miR-1281	41.93	105.42	219.79	−5.25	−2.09
miR-6780b-5p	29.45	28.84	155.42	−5.26	−5.38
miR-6069	10.48	24.25	56.10	−5.36	−2.31
miR-5001-5p	65.80	69.07	380.04	−5.77	−5.47
miR-6798-5p	17.75	7.26	103.25	−5.82	−14.15
miR-4706	209.38	219.79	1305.15	−6.26	−5.94
miR-6716-5p	6.32	5.21	39.95	−6.35	−7.68
miR-191-3p	105.42	97.01	670.92	−6.38	−6.92
miR-6893-5p	186.11	190.02	1200.98	−6.45	−6.32

miR-920	50.21	107.63	324.03	−6.45	−3
miR-4651	111.43	191.34	754.83	−6.74	−3.94
miR-1224-5p	46.53	51.63	321.80	−6.94	−6.25
miR-3154	31.78	103.97	230.72	−7.24	−2.22
miR-1231	16.91	47.84	131.60	−7.82	−2.77
miR-6813-5p	46.21	45.57	388.02	−8.42	−8.54
miR-5571-5p	10.20	19.16	86.82	−8.53	−4.52
miR-6754-5p	491.14	272.48	4211.15	−8.58	−15.44
miR-557	2.35	4.23	24.08	−10.21	−5.69
miR-4467	652.58	448.82	6936.54	−10.6	−15.39
miR-4430	11.96	29.04	144.01	−12.02	−4.98
miR-642b-3p	56.89	54.95	724.08	−12.73	−13.15
miR-6824-5p	206.50	826.00	2836.70	−13.65	−3.42
miR-6825-5p	4.79	17.88	91.77	−19.12	−5.13
miR-3937	2.95	3.78	60.97	−20.71	−16.19
miR-371b-5p	3.51	6.36	73.01	−20.88	−11.43
miR-572	7.11	45.89	174.85	−24.62	−3.82
miR-6068	23.26	66.26	600.49	−25.78	−9.03

Values shown are median intensities. Those miRNAs that were validated by qRT-PCR are depicted in bold type.

Table S4. List of top 100 differentially expressed genes between 786-O parental (P) and resistant clones c1 and c2. Arranged according to FDR *F-value*.

Gene	Clone#1	Clone#2	Parental	c1/P (Fold)	c2/P(Fold)	*p*-val
WLS	2091.03	903.89	20.97	99.73	43.11	2.47E-09
CTHRC1	26801.0	79023.8	67.65	396.18	1168.14	1.73E-08
RELN	625.99	317.37	15.14	41.36	20.97	8.57E-08
PQLC2L	474.41	45.57	20.39	23.26	2.23	8.76E-08
KCNIP1	12854.6	2628.46	27.67	464.65	95.01	1.07E-07
ANTXR1	21469.4	11746.6	48.84	439.59	240.52	2.72E-07
APOL2	3848.29	3304.00	41764	−10.85	−12.64	4.91E-07
DKK3	3956.48	5914.33	143.01	27.67	41.36	8.11E-07
EVI2A	156.50	1052.79	17.75	8.82	59.30	9.72E-07
DSC2	501.46	380.04	26.91	18.64	14.12	1.05E-06
PRODH	42.52	41.36	286.03	−6.73	−6.92	1.20E-06
COL12A1	1595.73	205.07	32.90	48.50	6.23	1.20E-06
ENPP3	67.65	64.00	670.92	−9.92	−10.48	1.43E-06
BORCS	22.63	45.25	304.44	−13.45	−6.73	1.59E-06
SEMA5A	149522	81245.4	2628.4	56.89	30.91	1.71E-06
DNAJC1	5996.90	4389.98	64.89	92.41	67.65	2.04E-06
GNG4	222.86	369.65	17.15	13.00	21.56	2.11E-06
CLMP	592.22	1468.37	68.12	8.69	21.56	2.34E-06
HERC5	290.02	296.11	17.88	16.22	16.56	2.57E-06
DMKN	50.56	256.00	20.53	2.46	12.47	2.73E-06
CA12	458.25	2179.83	69272	−151.17	−31.78	2.76E-06
SLC38A5	1924.14	26249.4	108.38	17.75	242.19	3.28E-06
AGA	1360.57	1060.11	292.04	4.66	3.63	4.43E-06
PSG5	102126	2256.70	48.84	2091.03	46.21	4.79E-06
CCDC80	22226.6	27939.1	84.45	263.20	330.84	5.04E-06
SFTA1P	448.82	131.60	36.50	12.30	3.61	5.33E-06
SERPIN2	1807.78	7281.40	430.54	4.20	16.91	5.54E-06
CHRDL1	14066.7	65083.3	209.38	67.18	310.83	6.70E-06
COL6A3	7590.61	38165.9	64.45	117.78	592.22	7.17E-06
ST6GL1	102.54	218.27	25.28	4.06	8.63	7.39E-06
VWDE	533.74	512.00	30.27	17.63	16.91	8.87E-06
AAMTS5	4299.64	2272.40	29.24	147.03	77.71	9.11E-06
RRAGD	2896.31	1710.26	24.59	117.78	69.55	9.13E-06
H1F0	280.14	110.66	40.79	6.87	2.71	9.44E-06
NOG	238.86	151.17	21.56	11.08	7.01	9.46E-06
ALPK3	15.45	16.45	51.27	−3.32	−3.12	9.51E-06
MYLK	4482.23	2702.35	317.37	14.12	8.51	9.53E-06
KLF8	109.90	42.22	11.31	9.71	3.73	1.06E-05
AS3MT	20.82	35.75	315.17	−15.14	−8.82	1.11E-05
BARX2	11.79	11.39	47.18	−4.00	−4.14	1.18E-05
TSPAN2	3169.41	744.43	116.16	27.28	6.41	1.21E-05
NPAS2	333.14	501.46	74.03	4.50	6.77	1.22E-05
RNF150	135.30	109.90	10.13	13.36	10.85	1.24E-05
HOXA13	60.97	123.64	26.35	2.31	4.69	1.32E-05
CRYAB	27939.1	1686.71	20.11	1389.16	83.87	1.39E-05
PDE1C	81.01	235.57	32.45	2.50	7.26	1.50E-05
LINC003	39.12	27.10	292.04	−7.46	−10.78	1.51E-05
MOXD1	8135.41	10226.3	568.10	14.32	18.00	1.54E-05
PTCH1	407.31	153.28	64.89	6.28	2.36	1.67E-05
TNS3	4737.79	6122.90	1136.2	4.17	5.39	2.06E-05
C1orf21	7332.05	5556.65	372.22	19.70	14.93	2.53E-05
SAA1	1243.34	98.36	25.63	48.50	3.84	2.73E-05
DZIP1	330.84	250.73	55.33	5.98	4.53	2.80E-05
PTHLH	613.11	292.04	12503	−20.39	−42.81	2.85E-05
CDR1	1734.13	1217.75	37.53	46.21	32.45	2.86E-05
SIX1	230.72	207.94	27.10	8.51	7.67	3.05E-05
ECHDC2	955.43	1105.13	3565.7	−3.73	−3.23	3.74E-05
ITGB3	130.69	158.68	24.25	5.39	6.54	3.90E-05
AJAP1	374.81	112.21	35.75	10.48	3.14	4.05E-05
PLPP3	100.43	367.09	15.14	6.63	24.25	4.15E-05
SLC16A4	58.49	76.64	2048.0	−35.02	−26.72	4.61E-05
TRAM2	15393.1	10960.3	744.43	20.68	14.72	5.11E-05
PPIC	9741.98	5442.30	1722.1	5.66	3.16	5.14E-05
PTPN20	18.64	14.62	101.83	−5.46	−6.96	5.34E-05
PRSS12	184.82	58.49	23.92	7.73	2.45	5.35E-05

PMP22	7281.40	19349.3	749.61	9.71	25.81	5.56E-05
SORT1	3902.01	1552.09	62.68	62.25	24.76	5.76E-05
TRIM22	82.14	53.45	661.68	−8.06	−12.38	5.79E-05
COL6A1	240.52	670.92	49.18	4.89	13.64	5.92E-05
BASP1	4608.24	5712.87	243.88	18.90	23.43	6.45E-05
SHISA9	304.44	229.13	56.89	5.35	4.03	6.61E-05
PTGFR	401.71	588.13	21.26	18.90	27.67	6.79E-05
ST3GAL6	87.43	93.70	32.67	2.68	2.87	6.92E-05
ACSM3	191.34	604.67	12854.	−67.18	−21.26	7.07E-05
CUX1	9216.48	12330.98	81810.5	−8.88	−6.63	7.35E-05
DLG5	68.59	50.21	176.07	−2.57	−3.51	7.67E-05
ADAMTS3	125.37	200.85	31.12	4.03	6.45	7.82E-05
AFAP1L1	45.89	30.48	11.31	4.06	2.69	7.84E-05
PID1	37.53	34.30	10.48	3.58	3.27	7.92E-05
RPS6KA2	393.44	203.66	56.10	7.01	3.63	7.99E-05
DHCR7	5404.70	3541.14	91.14	59.30	38.85	8.12E-05
BET1	1884.54	4640.29	9475.59	−5.03	−2.04	8.38E-05
PVRL1	138.14	73.52	10.27	13.45	7.16	8.42E-05
KCNIP3	467.88	132.51	18.90	24.76	7.01	9.16E-05
PIK3IP1	81.01	65.34	21.11	3.84	3.10	9.22E-05
NID2	1379.57	8364.13	73.01	18.90	114.56	9.45E-05
SEMA6A	630.35	786.88	35857.8	−56.89	−45.57	0.0001
PCSK5	56.10	125.37	1067.48	−19.03	−8.51	0.0001
KMO	133.44	207.94	1408.55	−10.56	−6.77	0.0001
PHKA2	29.24	31.78	290.02	−9.92	−9.13	0.0001
ANXA4	47314.67	30786.28	447026	−9.45	−14.52	0.0001
RPRD1B	401.71	501.46	1509.65	−3.76	−3.01	0.0001
UFSP2	362.04	286.03	86.22	4.20	3.32	0.0001
C1QTNF1	221.32	171.25	49.52	4.47	3.46	0.0001
PANK3	1992.00	1200.98	347.29	5.74	3.46	0.0001
PSG2	230.72	77.71	33.82	6.82	2.30	0.0001
DPYSL2	388.02	477.71	50.91	7.62	9.38	0.0001
HIF1A	365623.6	577715	27939.1	13.09	20.68	0.0001
ZNF318	6427.31	2005.85	357.05	18.00	5.62	0.0001

Table S5. List of top 100 differentially expressed genes between A498 parental (P) and resistant clones c1 and c2. Arranged according to FDR *F-value*.

Gene	Clone#1	Clone#2	Parental	c1/P (Fold)	c2/P(Fold)	*p*-val
FN1	18.51	19.70	661.68	−35.73	−33.42	3.76×10^{-5}
RHOB	10513.82	14766.09	657.11	16.02	22.45	0.0004
FAR2	1226.22	68.59	190.02	6.47	−2.77	0.002
TINAGL1	670.92	42.81	18.51	36.39	2.32	0.002
HEXA	12416.75	9475.59	143.01	86.76	66.46	0.002
SULT1A4	724.08	433.53	2817.11	−3.89	−6.51	0.0022
C1S	533.74	4513.40	20171.07	−37.8	−4.47	0.0057
SEC14L2	159.79	200.85	962.07	−6.03	−4.81	0.0057
BST2	306.55	240.52	47.50	6.46	5.07	0.0057
GLRXP	47.18	46.53	306.55	−6.48	−6.57	0.0059
ADGRG2	113.77	3213.66	259.57	−2.28	12.39	0.0059
CYR61	25709.25	13216.02	1060.11	24.22	12.5	0.0059
DNAJC15	17.15	17.39	114.56	−6.67	−6.6	0.0078
ANPEP	4770.75	401.71	24491.61	−5.15	−61.07	0.0083
DMKN	1097.50	781.44	75.58	14.51	10.39	0.0083
UBE2L6	7643.41	3666.02	955.43	8	3.84	0.0094
DOK4	526.39	401.71	2256.70	−4.3	−5.65	0.0109
TNS1	28.25	21.71	233.94	−8.29	−10.74	0.011
FAM111A	1458.23	4153.18	151.17	9.64	27.39	0.011
MRPS35	7383.04	1144.10	2628.46	2.81	−2.29	0.0111
MAL2	23331.64	17928.91	1243.34	18.7	14.37	0.0123
plylo	24.42	30.70	199.47	−8.21	−6.52	0.0164
PRKAR2A	16.91	22.32	88.03	−5.2	−3.96	0.0164
NPC2	21027.65	23331.64	4482.23	4.68	5.2	0.0164
ITGB3	7750.10	744.43	1734.13	4.47	−2.34	0.0167
N4BP1	955.43	831.75	2817.11	−2.96	−3.4	0.0168
TMEM139	1251.98	1746.20	149.09	8.42	11.74	0.0168
USH1C	38.59	72.00	484.38	−12.48	−6.72	0.0171
NAMPT	744.43	1370.04	5955.47	−8.05	−4.35	0.0171
PRKD3	80.45	17.63	280.14	−3.48	−15.94	0.0171
FCHSD2	3061.45	4067.71	448.82	6.82	9.1	0.0174
LINC0067	85.04	588.13	1951.00	−22.96	−3.34	0.0191
NEDD4L	2936.74	1499.22	481.04	6.14	3.12	0.0191
ST3GAL6	94.35	83.87	754.83	−8.02	−9.01	0.0196
ARHGAP1	2005.85	6122.90	13873.08	−6.92	−2.26	0.0196
MIR3654	198668.00	177812.4	55108.99	3.61	3.24	0.0196
SNAPC1	24154.43	5007.93	1698.45	14.22	2.95	0.0196
TTC9C	685.02	504.95	146.02	4.7	3.47	0.0222
SLC35F2	1448.15	354.59	159.79	9.03	2.21	0.0223
SERINC5	310.83	541.19	2076.59	−6.69	−3.83	0.0225
ETFB	2876.30	2019.80	9741.98	−3.39	−4.83	0.0225
ANKRD13	1097.50	2105.58	501.46	2.19	4.19	0.0225
PPFIBP1	13493.72	734.19	2759.13	4.89	−3.77	0.0225
GLRX	1234.75	2683.69	29532.18	−23.91	−10.95	0.0232
NOB1	3743.05	3326.99	13307.94	−3.55	−3.98	0.0239
WDR59	729.11	116.16	278.20	2.62	−2.39	0.0239
STX3	3929.15	4737.79	471.14	8.35	10.06	0.0239
SCIN	512.00	11190.60	52.35	9.81	213.89	0.0239
SLFN13	401.71	308.69	32.00	12.55	9.66	0.0239
VPS26B	6208.38	2683.69	1332.57	4.68	2.01	0.0247
PCED1B	86.22	218.27	749.61	−8.71	−3.42	0.0262
NUAK2	6888.62	3125.78	263.20	26.07	11.88	0.0262
ACKR3	317.37	3420.52	21769.19	−68.22	−6.35	0.0285
FGFR2	634.73	1686.71	72.00	8.79	23.46	0.0299
NAMPTP3	2005.85	6888.62	23331.64	−11.63	−3.37	0.0301
MAN1A1	182.28	171.25	885.29	−4.85	−5.17	0.0301
WASF3	222.86	168.90	33.13	6.74	5.1	0.0301
EPB41L2	11268.44	14664.09	1458.23	7.75	10.06	0.0301
DCLK1	265.03	136.24	24.08	11	5.65	0.0301
CNOT1	2574.36	2957.17	10884.59	−4.24	−3.68	0.0323
KAZN	130.69	65.80	20.39	6.38	3.23	0.0323
TPK1	8079.22	26068.14	1060.11	7.62	24.73	0.0338
RP11−24	3169.41	380.04	1176.27	2.69	−3.09	0.0365
AKR1E2	82.14	8.82	30.06	2.72	−3.41	0.0372
RHOD	1296.13	1176.27	240.52	5.37	4.86	0.0387

ZBTB40	243.88	160.90	68.59	3.55	2.34	0.039
CDK10	166.57	163.14	625.99	−3.76	−3.83	0.0407
ARL4C	3902.01	1160.07	11585.24	−2.97	−9.99	0.041
NRP1	2005.85	749.61	6208.38	−3.09	−8.32	0.0434
TNFAIP6	73.52	215.27	1951.00	−26.55	−9.04	0.0443
KYNU	128.89	333.14	1584.71	−12.36	−4.76	0.0444
CLMP	1530.73	274.37	6984.79	−4.56	−25.41	0.0467
ANKRD44	37.01	45.89	218.27	−5.92	−4.78	0.0477
FADS2	998913.34	985161.0	279018.2	3.59	3.53	0.0477
NAE1	922.88	648.07	2610.30	−2.83	−4.04	0.0483
EEF1A2	1820.35	3258.52	826.00	2.2	3.94	0.0483
SC5D	11268.44	3326.99	198.09	57.19	16.83	0.0483
KCNIP1	8659.09	652.58	91.14	94.62	7.13	0.0483
CALML4	165.42	261.38	1112.82	−6.74	−4.28	0.0502
SLC22A18S	263.20	572.05	1770.57	−6.7	−3.09	0.0544
STS	42.81	89.26	18.25	2.35	4.87	0.0544
HNRNPUL	1795.29	2385.37	634.73	2.84	3.77	0.0555
CSRP1	831.75	328.56	97.01	8.58	3.38	0.0555
CLU3	568.10	390.72	2385.37	−4.2	−6.1	0.0565
CLSTN1	1398.83	1871.53	410.15	3.41	4.59	0.0565
CHKA	1314.23	1009.90	128.00	10.2	7.85	0.0565
EZR	87076.75	106463	15716.5	5.58	6.82	0.058
RSPRY1	1418.35	1168.14	7858.29	−5.53	−6.68	0.0585
IL6	13969.57	744.43	198.09	70.63	3.76	0.0586
NQO1	197295.70	256749.1	746612.4	−3.78	−2.9	0.0602
SLX1	369.65	233.94	1097.50	−2.97	−4.69	0.0602
TMSB4X	40622.74	28924.41	5792.62	7.01	4.98	0.0602
PTGIS	65.80	580.04	252.48	−3.84	2.3	0.0606
RTN3	32316.87	56658.31	10660.59	3.03	5.29	0.061
CORO1B	526.39	685.02	222.86	2.36	3.08	0.0636
BAD	213.78	407.31	105.42	2.03	3.88	0.0643
MYO1D	8964.45	6746.86	552.56	16.22	12.22	0.0653
STARD10	776.05	781.44	215.27	3.6	3.63	0.0654
HEBP1	1675.06	2134.97	8364.13	−5	−3.93	0.0674
TRMT112	14462.21	16270.83	3258.52	4.44	5	0.0674

Table S6. List of top 100 differentially expressed genes between Caki−1 parental (P) and resistant clones c1 and c2. Arranged according to FDR *F−value*.

Gene	Clone#1	Clone#2	Parental	c1/P (Fold)	c2/P(Fold)	*p*−val
CA2	25.28	28.05	4240.45	−168.05	−151.18	2.44 × $^{-7}$
FGB	8.22	12.21	8719.32	1063.42	−713.71	2.52 × $^{-6}$
PFKFB3	265.03	53.82	3169.41	−11.93	−58.82	4.02 × $^{-6}$
TRHDE	25.81	8.57	278.20	−10.77	−32.5	4.26 × $^{-6}$
COLEC10	1002.93	18053	116502	−115.68	−6.46	1.07 × $^{-5}$
NR5A2	15.67	25.81	333.14	−21.22	−12.92	1.70 × $^{-5}$
AOAH	17.03	20.53	471.14	−27.68	−22.99	4.40 × $^{-5}$
SLC1A1	218.27	73.52	2120.22	−9.71	−28.94	4.40 × $^{-5}$
SLC34A2	16.45	13.09	2164.77	−131.7	−164.79	4.40 × $^{-5}$
ZPLD1	62.25	30.48	12077.2	−194.15	−394.76	5.71 × $^{-5}$
GDA	26.17	16.56	484.38	−18.57	−29.4	6.26 × $^{-5}$
DCDC2	146.02	162.02	3420.52	−23.5	−21.1	6.46 × $^{-5}$
ACSM3	41.07	45.25	333.14	−8.09	−7.34	9.92 × $^{-5}$
HAVCR1P1	643.59	294.07	10884.5	−16.97	−37.03	0.0001
ADD3	194.01	198.09	2194.99	−11.35	−11.11	0.0001
CD24P4	3717.20	1710.2	34397.2	−9.23	−20.07	0.0001
HSPA12A	29.24	51.63	200.85	−6.85	−3.87	0.0001
ADAMTS9	16.91	20.97	362.04	−21.51	−17.25	0.0001
IDH2	1428.22	458.25	6562.36	−4.6	−14.28	0.0001
ARRDC4	442.64	192.67	19.43	22.92	9.96	0.0002
HNF1B	675.59	430.54	11113.30	−16.39	−25.86	0.0002
KCNJ16	67.18	41.64	8248.98	−122.59	−198.35	0.0002
SULF2	1184.45	40.79	184.82	6.41	−4.53	0.0002
LAMA3	709.18	765.36	29328.18	−41.23	−38.33	0.0002
GUCY1B3	148.06	26.72	2005.85	−13.52	−74.79	0.0003
FHL1	38431.46	164.28	436.55	87.93	−2.66	0.0003
TM4SF18	34.06	26.54	451.94	−13.3	−17.14	0.0003
MLK4	48.17	119.43	1618.00	−33.45	−13.47	0.0003
EPCAM	17.63	28.25	873.10	−49.23	−30.74	0.0003
LRRN1	56658.31	401.71	69.07	820.71	5.8	0.0003
SYT14	657.11	1478.8	75.06	8.71	19.61	0.0004
HAVCR1	28526	23170	762300	−26.81	−33.06	0.0004
HBEGF	3666.	424.61	1234.75	2.97	−2.91	0.0004
SLC4A4	39.12	30.91	568.10	−14.49	−18.36	0.0004
AGT	85.63	15.78	427.57	−4.98	−27.07	0.0004
LAMP3	1217.7	7538.8	221.32	5.5	34.13	0.0004
ARHGAP29	3640.7	6746.8	32995.9	−9.05	−4.9	0.0005
ERICH2	372.22	1675	131.60	2.82	12.71	0.0005
SPP1	11036	15286	386470.	−35.17	−25.33	0.0005
PDE1A	367.09	32.45	170.07	2.15	−5.25	0.0005
GUCY1A3	11.31	12.91	230.72	−20.41	−17.97	0.0005
CXXC5	315.17	159.79	1418.35	−4.48	−8.85	0.0006
C1QTNF1	352.14	280.14	50.56	6.95	5.54	0.0006
GPNMB	38967	9864	4153.18	9.4	23.86	0.0006
EFHB	12.13	18.51	109.14	−9.04	−5.93	0.0006
MACROD	35.51	24.93	170.07	−4.8	−6.81	0.0006
ANKRD29	137.19	176.07	1398.83	−10.2	−7.98	0.0006
OSTM1	25006	20738	4299.64	5.82	4.84	0.0006
SC5D	25321	68794	1595.73	158.5	43.24	0.0006
NOV	143.01	522.76	31.56	4.53	16.49	0.0006
PDE4D	26.17	224.41	1097.50	−42.13	−4.9	0.0006
KIF12	6.68	9.06	30.48	−4.54	−3.35	0.0007
HGD	49.87	35.02	704.28	−14.17	−20.13	0.0007
PARD6B	3040.30	4124.4	14766.0	−4.88	−3.59	0.0007
RCAN1	8248.98	2998.5	491.14	16.84	6.12	0.0008
MLLT3	349.71	982.29	8023.41	−22.93	−8.12	0.0008
ADGRG1	23010.4	2916.4	82952.6	−3.6	−28.43	0.0008
AMIGO2	47.84	56.10	564.18	−11.83	−10.04	0.0008
PDE5A	2134.97	407.31	31.56	67.79	12.89	0.0008
SCG5	560.28	23.10	122.79	4.58	−5.31	0.0008
UGT2B7	229.13	128.89	2179.83	−9.49	−16.9	0.0009
MOCOS	96.34	2486.6	809.00	−8.36	3.08	0.001

CYTH3	12245.8	8719.3	1226.22	10.02	7.13	0.001
CTPS1	6517.03	23331	2320.15	2.81	10.03	0.001
CRABP1	16.00	14.03	51.63	−3.24	−3.69	0.001
NUPR1	87076.7	13569	26068.1	3.34	5.21	0.001
TBL1XR1	382.68	442.64	4451.27	−11.69	−10.1	0.0011
FUOM	99.73	51.27	342.51	−3.44	−6.68	0.0011
EIF3E	41476.3	52498.	200049	−4.82	−3.8	0.0011
TRPC6	75.06	45.57	272.48	−3.62	−5.94	0.0011
ARMC9	4608.24	3640.0	729.11	6.34	5	0.0011
HMGA2	144.01	149.09	1045.52	−7.23	−6.99	0.0012
SLC17A1	22.63	53.45	398.93	−17.71	−7.47	0.0012
SAMD5	362.04	44.63	19.70	18.34	2.25	0.0012
OR51B5	64.00	99.04	1341.84	−21.04	−13.56	0.0012
CD24	100024	51063.	565826.	−5.66	−11.11	0.0013
ANXA13	15.67	14.42	124.50	−7.96	−8.65	0.0013
MYO1E	2610.30	445.72	5595.30	−2.15	−12.59	0.0013
GGT8P	35.26	19.29	75.06	−2.14	−3.89	0.0013
A−AS1	5480.15	20031	43538.3	−7.93	−2.17	0.0013
KAT6B	955.43	1192.6	3590.58	−3.77	−3.02	0.0013
DSC3	31.78	48.17	261.38	−8.17	−5.41	0.0013
MKRN9P	95950.3	4608.2	28526.2	3.37	−6.2	0.0013
TNIK	43.11	58.49	3492.39	−81.17	−59.9	0.0013
CAMLG	962.07	1562.8	3795.30	−3.93	−2.43	0.0014
PTPN13	1192.69	1710.2	13493.7	−11.35	−7.93	0.0015
AK4	116.97	119.43	897.64	−7.65	−7.5	0.0015
RPL12P12	4482.23	5148.7	18053.6	−4.01	−3.51	0.0015
ANXA4	12854.6	12944.	80684.2	−6.26	−6.24	0.0016
ARHGAP1	2957.17	2936.7	12765.8	−4.3	−4.32	0.0016
VCAM1	1144.10	63743	313911	−273.14	−4.89	0.0016
ANPEP	154.34	24.93	1097.50	−7.1	−44.02	0.0016
PALLD	4640.29	792.35	1595.73	2.91	−2.02	0.0016
CFAP70	128.89	121.94	25.63	5.06	4.76	0.0016
CCL28	304.44	66.72	24.25	12.53	2.75	0.0016
LIMA1	33456.5	45073.	3875.05	8.65	11.58	0.0016
CECR2	8.34	10.85	45.89	−5.5	−4.24	0.0016
DAB2	2149.82	613.11	4389.98	−2.04	−7.18	0.0017
SCG5	439.59	22.63	149.09	2.96	−6.58	0.0017

Table S7. List of significantly enriched KEGG pathways for 786−O cell line.

**# Genes (Genes in Pathway)**	**Description**	**Genes**	***p*−value**
10 (149)	MicroRNAs in cancer	*HOXD10*丨*NOTCH2*丨*MMP16*丨*ITGA5*丨*PTGS2*丨*PTEN*丨*RECK*丨*FSCN1*丨*CDKN1A*丨*ITGB3*	4.09 ×10 $^{-9}$
8 (195)	Proteoglycans in cancer	*MMP2*丨*HOXD10*丨*ITGA5*丨*RAC1*丨*MSN*丨*CDKN1A*丨*HIF1A*丨*ITGB3*	1.61 × 10^{-5}
10 (515)	Pathways in cancer	*MMP2*丨*NOTCH2*丨*LRP6*丨*EPAS1*丨*SP1*丨*RAC1*丨*PTGS2*丨*PTEN*丨*CDKN1A*丨*HIF1A*	3.34 × 10^{-4}
8 (317)	Human papillomavirus infection	*TBPL1*丨*NOTCH2*丨*ITGA5*丨*PTGS2*丨*PTEN*丨*PRKCZ*丨*CDKN1A*丨*ITGB3*	5.52 × 10^{-4}
4 (63)	Mitophagy − animal	*SP1*丨*BECN1*丨*BNIP3L*丨*HIF1A*	4.90 × 10^{-4}
4 (68)	Renal cell carcinoma	*EPAS1*丨*RAC1*丨*CDKN1A*丨*HIF1A*	6.51 × 10^{-3}
5 (147)	Breast cancer	*NOTCH2*丨*LRP6*丨*SP1*丨*PTEN*丨*CDKN1A*	9.33 × 10^{-3}
4 (98)	Choline metabolism in cancer	*SP1*丨*WASF3*丨*RAC1*丨*HIF1A*	2.50 × 10^{-3}
5 (183)	Kaposi's sarcoma−associated herpesvirus infection	*RAC1*丨*BECN1*丨*PTGS2*丨*CDKN1A*丨*HIF1A*	2.50 × 10^{-3}
4 (95)	Endocrine resistance	*MMP2*丨*NOTCH2*丨*SP1*丨*CDKN1A*	2.20 × 10^{-3}
6 (348)	PI3K−Akt signaling pathway	*ITGA5*丨*RAC1*丨*PTEN*丨*CDKN1A*丨*BDNF*丨*ITGB3*	6.70 × 10^{-3}
4 (133)	Fluid shear stress and atherosclerosis	*MMP2*丨*RAC1*丨*PRKCZ*丨*ITGB3*	7.70 × 10^{-3}

Table S8. List of significantly enriched KEGG pathways for A498 cell line.

# Genes (Genes in Pathway)	Description	Genes	*p*−value
2 (87)	Gap junction	*EGFR*|*ITPR1*	3.00×10^{-3}
2 (88)	GnRH signaling pathway	*EGFR*|*ITPR1*	3.10×10^{-3}
2 (133)	Estrogen signaling pathway	*EGFR*|*ITPR1*	7.00×10^{-3}
2 (133)	Apelin signaling pathway	*ITPR1*|*CTGF*	7.00×10^{-3}
2 (149)	Proteoglycans in cancer	*EGFR*|*ITPR1*	8.70×10^{-3}
2 (153)	PI3K−Akt signaling pathway	*EGFR*|*ITPR1*	9.20×10^{-3}

Table S9. List of significantly enriched KEGG pathways for Caki−1 cell line.

# Genes (Genes in Pathway)	Description	Genes	*p*−value
5 (133)	Fluid shear stress and atherosclerosis	*KLF2*|*ITGAV*|*ICAM1*|*CAV1*|*ITGB3*	9.34×10^{-6}
4 (115)	Thyroid hormone signaling pathway	*CCND1*|*ITGAV*|*SLC2A1*|*ITGB3*	2.90×10^{-5}
3 (56)	Viral myocarditis	*CCND1*|*ICAM1*|*CAV1*	1.71×10^{-3}
4 (195)	Proteoglycans in cancer	*CCND1*|*ITGAV*|*CAV1*|*ITGB3*	2.22×10^{-3}
4 (197)	Focal adhesion	*CCND1*|*ITGAV*|*CAV1*|*ITGB3*	2.31×10^{-3}
4 (317)	Human papillomavirus infection	*CCND1*|*IRF1*|*ITGAV*|*ITGB3*	1.40×10^{-3}
3 (133)	Apelin signaling pathway	*CCND1*|*KLF2*|*CTGF*	2.10×10^{-3}
3 (149)	MicroRNAs in cancer	*CCND1*|*DNMT3B*|*ITGB3*	2.90×10^{-3}
3 (152)	Hippo signaling pathway	*CCND1*|*CTGF*|*BMP2*	3.00×10^{-3}
4 (515)	Pathways in cancer	*CCND1*|*ITGAV*|*BMP2*|*SLC2A1*	8.80×10^{-3}

Table S10. List of primary and secondary antibodies used in the Western Blots. All antibodies were diluted in 5% non−fat dry milk in TBS−T.

Anti−	Cat. No.	Dilution	Manufacturer
HIF−1α	610958	1:250	BD Transduction Laboratories
HIF−2α	sc−46691	1:500	Santa Cruz
PD−L1	#13684	1:500	Cell Signaling Technologies
Cyclin D1	ab16663	1:500	Abcam
β−actin	A5441	1:5000	Sigma
α−tubulin	ab52866	1:1000	Abcam
Mouse, IgG−HRP	sc−2005	1:2000	Santa Cruz
Rabbit, IgG−HRP	sc−2357	1:2000	Santa Cruz
